# LAMTOR2 regulates dendritic cell homeostasis through FLT3-dependent mTOR signalling

**DOI:** 10.1038/ncomms6138

**Published:** 2014-10-22

**Authors:** Julia M. Scheffler, Florian Sparber, Christoph H. Tripp, Caroline Herrmann, Alexandra Humenberger, Johanna Blitz, Nikolaus Romani, Patrizia Stoitzner, Lukas A. Huber

**Affiliations:** 1 Biocenter, Division of Cell Biology, Innsbruck Medical University, Innrain 80-82, Innsbruck A6020, Austria; 2 Department of Dermatology and Venereology, Innsbruck Medical University, Innsbruck A6020, Austria; 3 Austrian Drug Screening Institute (ADSI), Innsbruck A6020, Austria

## Abstract

The receptor tyrosine kinase Flt3 and its ligand are crucial for dendritic cell (DC) homeostasis by activating downstream effectors including mammalian target of Rapamycin (mTOR) signalling. LAMTOR2 is a member of the Ragulator/LAMTOR complex known to regulate mTOR and extracellular signal-regulated kinase activation on the late endosome as well as endosomal biogenesis. Here we show in mice that conditional ablation of *LAMTOR2* in DCs results in a severe disturbance of the DC compartment caused by accumulation of Flt3 on the cell surface. This results in an increased downstream activation of the AKT/mTOR signalling pathway and subsequently to a massive expansion of conventional DCs and plasmacytoid DCs in ageing mice. Finally, we can revert the symptoms *in vivo* by inhibiting the activation of Flt3 and its downstream target mTOR.

## Abstract (web summary)

LAMTOR2 is involved in mTOR and ERK signalling and plays a role in immunity, but its function in dendritic cells (DCs) is not clear. Here the authors show that deletion of LAMTOR2 in DCs results in increased mTOR signalling, accumulation of Flt3 on the cell surface and excessive DC proliferation in ageing mice.

## Graphical abstract

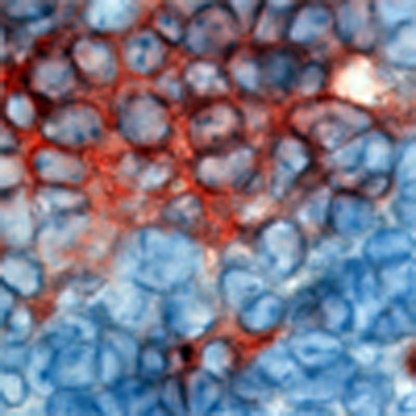

Cells use signalling cascades to translate extracellular information into specific downstream responses. A major signalling pathway regulating cell growth, differentiation and survival is the extracellular signal-regulated kinase (ERK) cascade, which belongs to the family of mitogen-activated protein kinase (MAPK) pathways. To ensure specific signalling, a spatial and temporal segregation must be achieved. After activation, receptors are endocytosed and transferred to late endosomes for degradation. However, it was shown that late endosomes also function as signalling platforms. There, the late endosomal/lysosomal adaptor and MAPK and mammalian target of Rapamycin (mTOR) activator (LAMTOR) complex serves as a convergence point for ERK and mTOR complex 1 (mTORC1) signalling. It consists of LAMTOR1 (p18), LAMTOR2 (p14), LAMTOR3 (MP1), LAMTOR4 (HBXIP) and LAMTOR5 (C7orf59)^1^,^2^,^3^,^4^,^5^,^6^,^7^,^8^. Deletion of *LAMTOR2* results in a destabilization and cytosolic mislocalization of the remaining complex components^5^,^9^. In addition, conditional gene ablation of *LAMTOR2* in keratinocytes in the epidermis of mice revealed its importance for tissue homeostasis, cellular proliferation and endosomal traffic^10^. A previously identified human primary immunodeficiency syndrome was ascribed to a point mutation in the *LAMTOR2* gene causing a hypomorph allele and reduced protein levels of LAMTOR2. Those patients have severe immunological defects affecting the innate and adaptive immunity, which can be related to a disturbed endosomal- and lysosomal biogenesis. They suffer from neutropenia, defects in T-cell function and B-cell maturation and subsequently have recurrent broncho-pulmonary infections^11^. In correlation with these observations, we could recently show in a mouse model that LAMTOR2 is crucial for macrophages to fight *Salmonella* infection by controlling replication in the phagosome^12^. Based on these findings, we were interested in the role of LAMTOR2 for adaptive immunity.

Dendritic cells (DCs) are the initiators of adaptive immunity. Their ability to take up, process and finally present pathogenic as well as self-antigens to T cells, is strictly dependent on efficient late endosomal-biogenesis^13^,^14^. DCs originate from haematopoietic stem cells and differentiate via common progenitors to so-called pre-DCs, which finally seed various organs to become fully differentiated DCs. Specific cytokine signals are indispensable throughout this development as well as for the homeostasis of DCs^15^. Originally, it was thought that granulocyte-macrophage colony-stimulating factor (GM-CSF) is the major cytokine promoting DC differentiation, as it allowed for the first time the *in vitro* generation of DCs from human blood and mouse bone marrow (BM)^16^,^17^,^18^. However, the discovery that mice lacking GM-CSF or its receptor still develop normal DC populations in the spleen and lymph nodes (LNs)^19^ led to the conclusion that GM-CSF is dispensable for steady-state DC development. As shown recently, this also holds true for differentiation of inflammatory DCs. In contrast, deletion or inhibition of another cytokine receptor, named Fms-like tyrosine kinase 3 ligand receptor (Flt3) and its ligand (Flt3-L), resulted in a tenfold reduction of plasmacytoid DCs (pDCs) and tissue resident DCs^20^,^21^. Conversely, injection of Flt3-L in mice increased DC numbers of various subtypes in many organs^22^. These findings together with the fact that Flt3 is expressed on common DC progenitors (CDPs), pre-DCs and their progeny^23^ underline the importance of Flt3 receptor signalling for DC differentiation. However, little was known about the downstream Flt3 signalling controlling DC development until recent findings showed that the mammalian target of Rapamycin (mTOR) plays a major role in this signalling cascade. It was shown that the phosphoinositide 3-kinase (PI3K)-AKT-mTOR signalling cascade downstream of Flt3 controls DC development and expansion^24^. Inhibiting this signalling pathway by Rapamycin resulted in an impairment of steady-state DC generation *in vivo*^25^.

As LAMTOR2, as member of the Ragulator/LAMTOR complex, is crucial not only for ERK but also for mTOR activation and influences endosomal biogenesis and receptor trafficking, we decided to investigate its function specifically in DCs. Therefore, we made use of a conditional knockout mouse model in which *LAMTOR2* can be specifically deleted in CD11c^+^ DCs^26^. Here we show that genetic ablation of *LAMTOR2* in DCs results in the accumulation of the Flt3-receptor on the plasma membrane accompanied by a deregulation of LAMTOR complex-mediated downstream signalling. As a consequence, late endosomal ERK signalling is abolished. However, despite the loss of the LAMTOR complex, ligand-induced AKT/mTORC1 signalling downstream of the Flt3 receptor is unexpectedly increased. The outcome of this enhanced mTOR signalling is an expansion of pDCs and conventional DCs (cDCs), which finally cause a myeloid proliferative syndrome in ageing mice.

Thus, we present evidence that LAMTOR2 is crucial for Flt3-dependent DC homeostasis and additionally describe new aspects of LAMTOR complex-mediated late endosomal signalling in immunity.

## Results

### Deletion of *LAMTOR2* in DCs alters DC homeostasis

**Figure 1:**
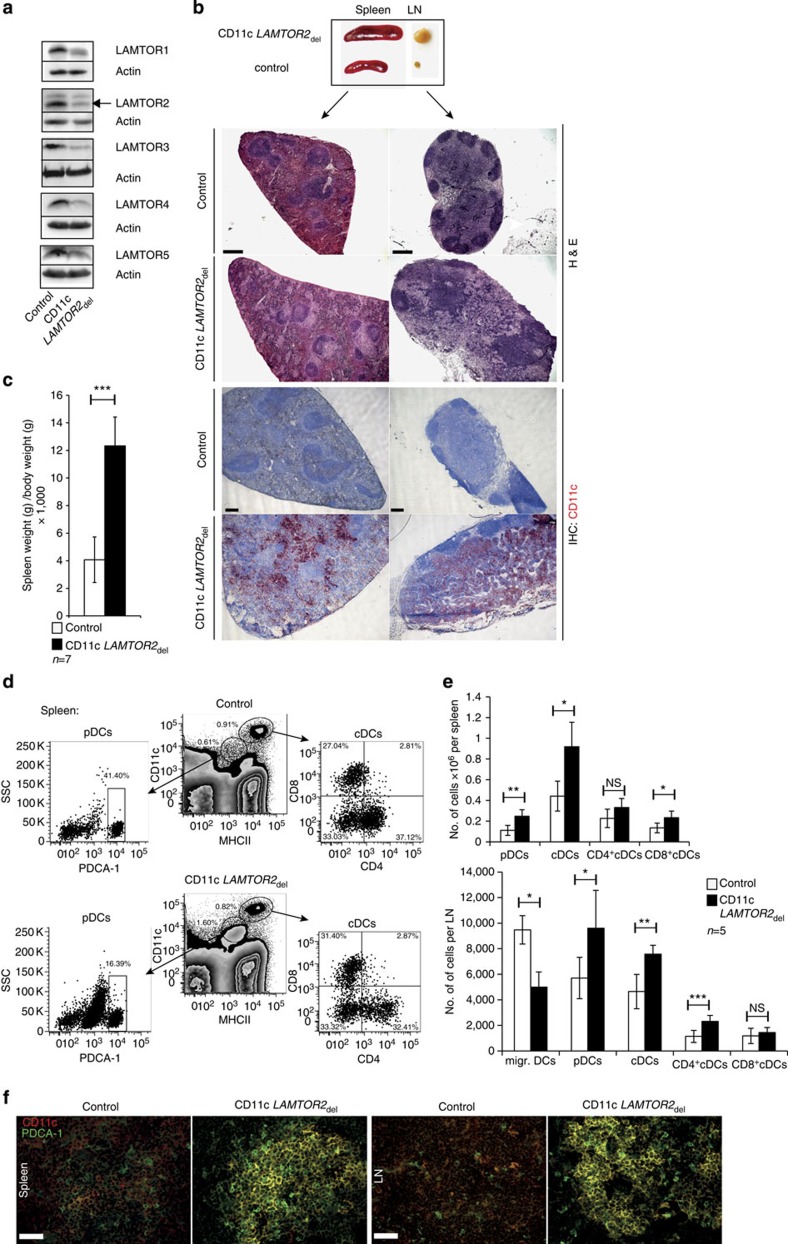
LAMTOR2 deficiency disturbed DC homeostasis. (**a**) Western blot of Flt3-L-BMDC cell lysates from control and CD11c *LAMTOR2*_del_ mice was performed against LAMTOR1 to LAMTOR5. Actin was used as a loading control. (**b**) Representative example of spleen and LNs from control and DC-specific *LAMTOR2*-deficient animals. Paraffin sections of spleen and LN from control and CD11c *LAMTOR2*_del_ mice were stained with H&E. Upper panels show an example of a spleen from a control and a CD11c *LAMTOR2*_del_ animal. The lower panels show the respective LNs. Arrowheads indicate infiltrates. Scale bar, 200 μm. Immunohistochemistry for the expression of CD11c was performed on spleen and LN cryosections of control and deficient animals and counterstained with haematoxylin (blue). Spleen sections are depicted in the upper panel; LN sections in the lower panel. Animals were at the age of 2–3 months. (**c**) Spleen weight in correlation to body weight was measured (*n*=7 per genotype), mean±s.d., ***P<0.001 as determined by unpaired Student’s *t*-test. (**d**,**e**) Analysis of DC populations in the spleen by flow cytometry of mice at the age of 3 months. One representative out five is shown. (**e**) Combined data of DC subset analyses of spleen and LN from five individually analysed mice per genotype are shown in cell numbers. Mean±s.d., *ns P>*0.05, *P<0.05, **P<0.01, ***P<0.001 as determined by unpaired Student’s *t*-test. (**f**) Cryosections of the spleen and LN of both genotypes were stained for CD11c (red) and PDCA-1 (green). Scale bar, 50 μm.

To specifically delete *LAMTOR2* in DCs of mice, we used a conditional mouse model in which the Cre recombinase is expressed under the promotor of the *CD11c* gene^26^. These mice were crossed with the *LAMTOR2*_f/f_ mice^10^ to obtain CD11c *LAMTOR2*_del_ mice harbouring the homozygous deletion of *LAMTOR2*. This could be confirmed via genotyping (Supplementary Fig. 1a), as well as on protein (Fig. 1a and Supplementary Fig. 1b) and mRNA levels (Supplementary Fig. 1c). In addition, a reduction of the protein levels of all other known LAMTOR components was observed (Fig. 1a), as described before^9^. To exclude an effect of background expression of Cre in other major immune cell compartments^26^, mRNA levels of LAMTOR2 in sorted splenic B and T cells were assessed (Supplementary Fig. 1c). No significant reduction was measured. Heterozygous CD11c *LAMTOR*2_del/+_ mice, possessing one functional *LAMTOR2* allele, were always used as control mice. CD11c *LAMTOR2*_del_ mice developed normally and were born in the expected Mendelian frequencies. Body weight (Supplementary Fig. 1d) and appearance of CD11c *LAMTOR2*_del_ mice were similar to control mice. However, at 3 months of age, the deficient mice displayed significantly enlarged spleens and LNs (Fig. 1b,c). Further analysis of these two organs revealed severe morphological alterations in tissue architecture. Histological staining with haematoxylin and eosin (H&E) of the spleen revealed a disturbed segregation of red and white pulpa, with increased germinal centres size and accumulations of infiltrating leukocytes (Fig. 1b, upper panel, arrow heads). The LNs of the CD11c *LAMTOR2*_del_ animals showed a similar morphology. B-cell areas also showed enlarged germinal centres and the in the T-cell zone massive infiltrates were found (Fig. 1b, lower panel, arrow heads). Immunohistochemistry for the expression of the DC marker CD11c showed that the cellular infiltrates largely consisted of CD11c^+^ DCs (Fig. 1b). Further histological investigation revealed infiltrates also in other organs of CD11c *LAMTOR2*_del_ mice. Conditional *LAMTOR2*-deficient mice developed massive infiltrates in the perivascular region of the liver starting at the age of 3 months (Supplementary Fig. 1e). To further characterize the infiltrating cells in the liver, immunofluorescence staining of cryosections was performed. The infiltrates were positive for the DC marker CD11c (Supplementary Fig. 1e) but negative for Gr-1 and F4-80 (data not shown).

**Figure 2:**
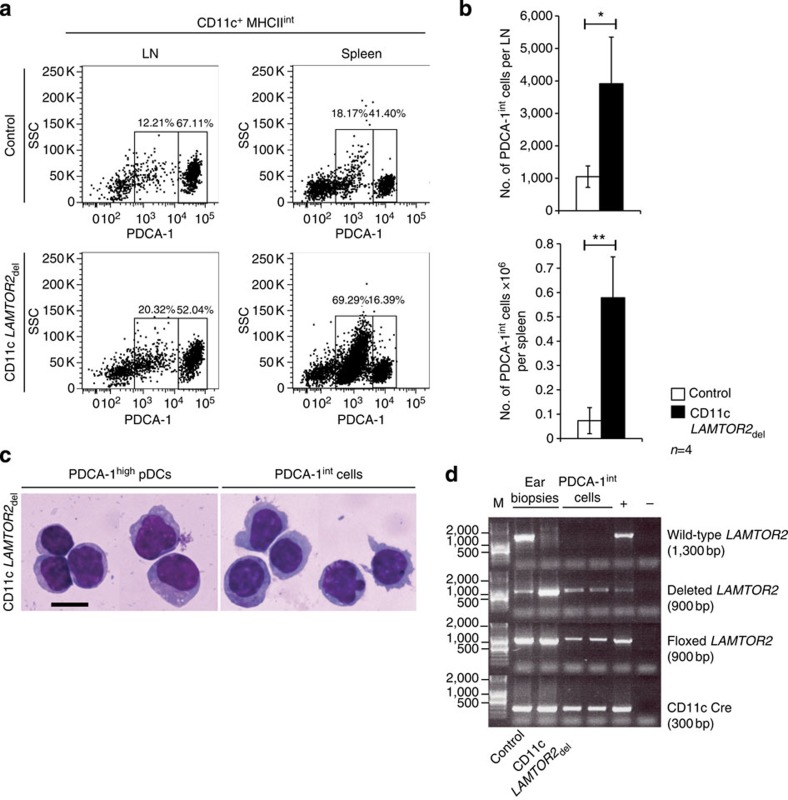
Expansion of CD11c^+^PDCA-1^int^ cells. (**a**,**b**) Analysis of PDCA-1^int^ population in the LN and spleen. One representative example is shown. (**b**) Combined analyses for both organs are shown in cell numbers. Four mice per genotype at the age of 3 months were analysed. Mean±s.d., *P<0.05, **P<0.01 as determined by unpaired Student’s *t*-test. (**c**) Morphological analysis of pDCs and PDCA-1^int^ cells were performed. Cells of a knockout animal were sorted and cytospins followed by a Pappenheim-staining were performed. Scale bar, 10 μm. (**d**) Sorted PDCA-1^int^ cells were genotyped for *LAMTOR2* deletion.

As disturbances in cellular populations leading to enlargements of the spleen and LNs of CD11c *LAMTOR2*_del_ animals were detected, flow cytometry analysis was performed. No significant alterations of cell numbers in major immune cell compartments including CD19^+^ B cells, CD3^+^ T cells and CD3^−^ Nkp46^+^ NK cells were observed (Supplementary Fig. 2a–d). However, major shifts in the DC compartment were revealed. In both lymphoid organs, a significant increase in numbers of PDCA-1^high^ pDCs and CD11c^high^ MHCII^high^ cDCs were measured (Fig. 1d,e). The reduction of migratory DCs in the skin draining LN of the CD11c *LAMTOR2*_del_ mice was described before^27^. To further confirm the DC expansion in those organs, immunofluorescence staining for CD11c and PDCA-1 was performed and underlined the described results (Fig. 1f). Interestingly, especially in the spleen, a population of CD11c^+^ MHCII^int^ and PDCA-1^int^ cells expanded significantly (Fig. 2a,b). Those cells exhibited a similar morphology as pDCs (Fig. 2c) and were also deficient for *LAMTOR2* (Fig. 2d).

### *LAMTOR2* deletion influences DC-dependent immunity in the spleen

**Figure 3:**
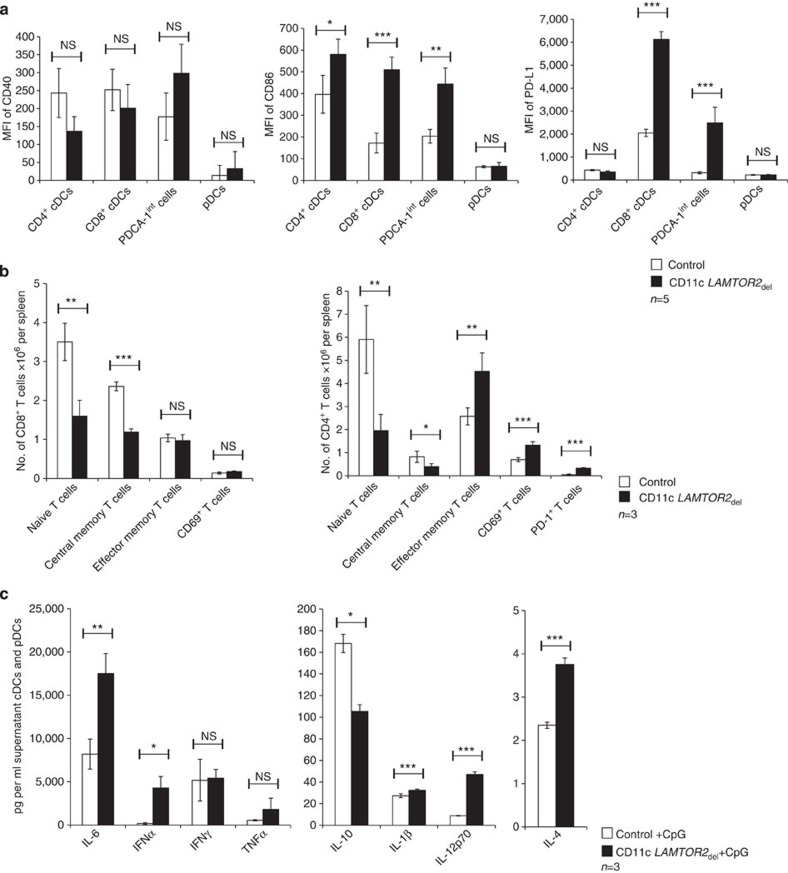
Characterization of the DC and T-cell compartment. (**a**) Surface expression of CD40, CD86 and PD-L1 on DC populations was measured by flow cytometry. Five control and five CD11c *LAMTOR2*_del_ mice were analysed. Mean±s.d., *ns: P>*0.05, *P<0.05, **P<0.01, ***P<0.001 as determined by unpaired Student’s *t*-test. (**b**) T-cell subsets of the spleen of control and CD11c *LAMTOR2*_del_ mice were analysed by flow cytometry. The left graph shows the distribution of CD8^+^ T-cell subsets as indicated (*n*=3). The right graph shows CD4^+^ T-cell subsets (*n*=4). Cells were pre-gated for CD4 and CD8 as indicated in Supplementary Fig. 2a. Mean±s.d., *ns: P>*0.05, *P<0.05, **P<0.01, ***P<0.001 as determined by unpaired Student’s *t*-test. (**c**) Dendritic cells were isolated of the spleen of control and CD11c *LAMTOR2*_del_ mice and stimulated with 2 μg ml^−1^ CpG. The indicated cytokines were measured with a multiplexing system in the supernatant (*n*=3). Mean±s.d., *ns: P>*0.05, *P<0.05, **P<0.01, ***P<0.001 as determined by unpaired Student’s *t*-test.

In a next step, we further characterized the activation status of the expanded DC populations. The expression of co-stimulatory molecules on cDC, pDC and on the PDCA-1^int^ cells was analysed. We observed increased expression of CD86 and PD-L1 on cDCs and the PDCA-1^int^ cells of CD11c *LAMTOR2*_del_ mice, whereas CD40 levels were not significantly changed. In general, pDCs showed a low expression of all three activation markers and no differences between control and *LAMTOR2*-deficient cells were detected (Fig. 3a). As this enhanced DC activation could also lead to T-cell stimulation, we analysed the T-cell compartment for common activation markers. In the CD8^+^ T-cell compartment of CD11c *LAMTOR2*_del_ mice, a reduction of naïve and central memory T cells was observed; however, effector memory T cells and the overall CD69 expression were unchanged. PD-1 expression was not detected on the CD8^+^ T cells. In the CD4^+^ T-cell compartment of CD11c *LAMTOR2*_del_ mice, a shift towards effector memory cells and increased expression of activation markers like CD69 and PD-1 was measured (Fig. 3b). These higher numbers of activated T cells in combination with increased CD86 and PD-L1 levels on DCs indicated induction of inflammation. To confirm these findings, we analysed the serum of control and CD11c *LAMTOR2*_del_ mice for inflammatory cytokines. There were no significant increases in interleukin (IL)-1β, IL-4 and IL-6, except for tumour-necrosis factor-α levels in the serum of CD11c *LAMTOR2*_del_ mice (Supplementary Fig. 3a). However, when we analysed the culture supernatant of total spleen cells, more pro-inflammatory cytokines, like IL-1β, IL-6 and tumour-necrosis factor-α, were secreted by CD11c *LAMTOR2*_del_ splenic cells. In addition, we also measured an elevation of IL-4, IL-10, interferon (IFN)-γ and IL-12p70, cytokines released by DCs and T cells (Supplementary Fig. 3b).

Next, we wondered if *LAMTOR2-*deficient DCs were more efficient T-cell stimulators. However, DCs from CD11c *LAMTOR2*_del_ mice, pulsed with ovalbumin protein, were equally potent in stimulating proliferation of ovalbumin-specific CD4^+^ and CD8^+^T cells in *in vitro* T-cell assays (data not shown).

Finally, we wanted to know how *LAMTOR2*-deficient DCs behave after Toll-like receptor stimulation with CpG *in vitro*. *LAMTOR2*-deleted cDCs and pDCs secreted increased amounts of IFN-α, IL-12p70, IL-4, IL-6 and IL1-β in response to Toll-like receptor stimulation. Interestingly, IL-10 levels were reduced (Fig. 3c).

Together, these findings indicated that *LAMTOR2*-deleted DCs showed some signs of activation leading to a local inflammatory process that also affected the T-cell compartment.

### DC expansion is due to increased proliferation

**Figure 4:**
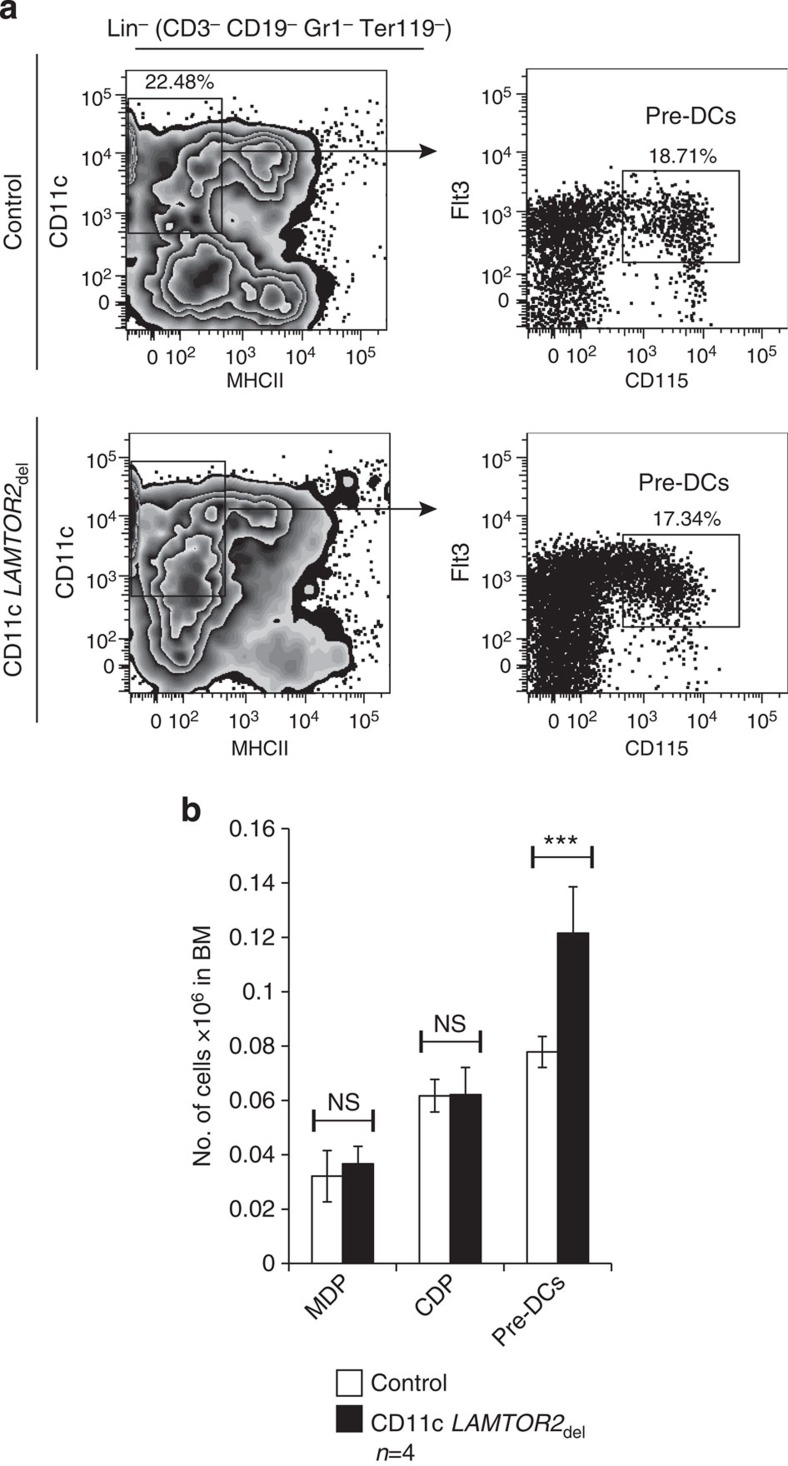
Increased numbers of pre-DCs in the bone marrow (BM). (**a**) Analysis of pre-DCs in BM samples of control and CD11c *LAMTOR2*_del_ mice. One representative of both genotypes is shown. Mice were 3 months of age. (**b**) Combined analysis of pre-DCs, CDPs and MDPs of four individual measurements for both genotypes are shown. Mean±s.d., *ns P>*0.05, ***P<0.001 as determined by unpaired Student’s *t*-test.

To assess whether DC homeostasis in CD11c *LAMTOR2*_del_ mice was already affected during differentiation, we analysed the macrophage and DC progenitors (MDPs), the common DC progenitors (CDPs) and the pre-DCs^28^ in the BM. We could not observe any differences for MDP or CDP numbers of both genotypes (Fig. 4b and Supplementary Fig. 4a). The pre-DC numbers, however, were significantly increased in the CD11c *LAMTOR2*_del_ mice (Fig. 4a,b).

**Figure 5:**
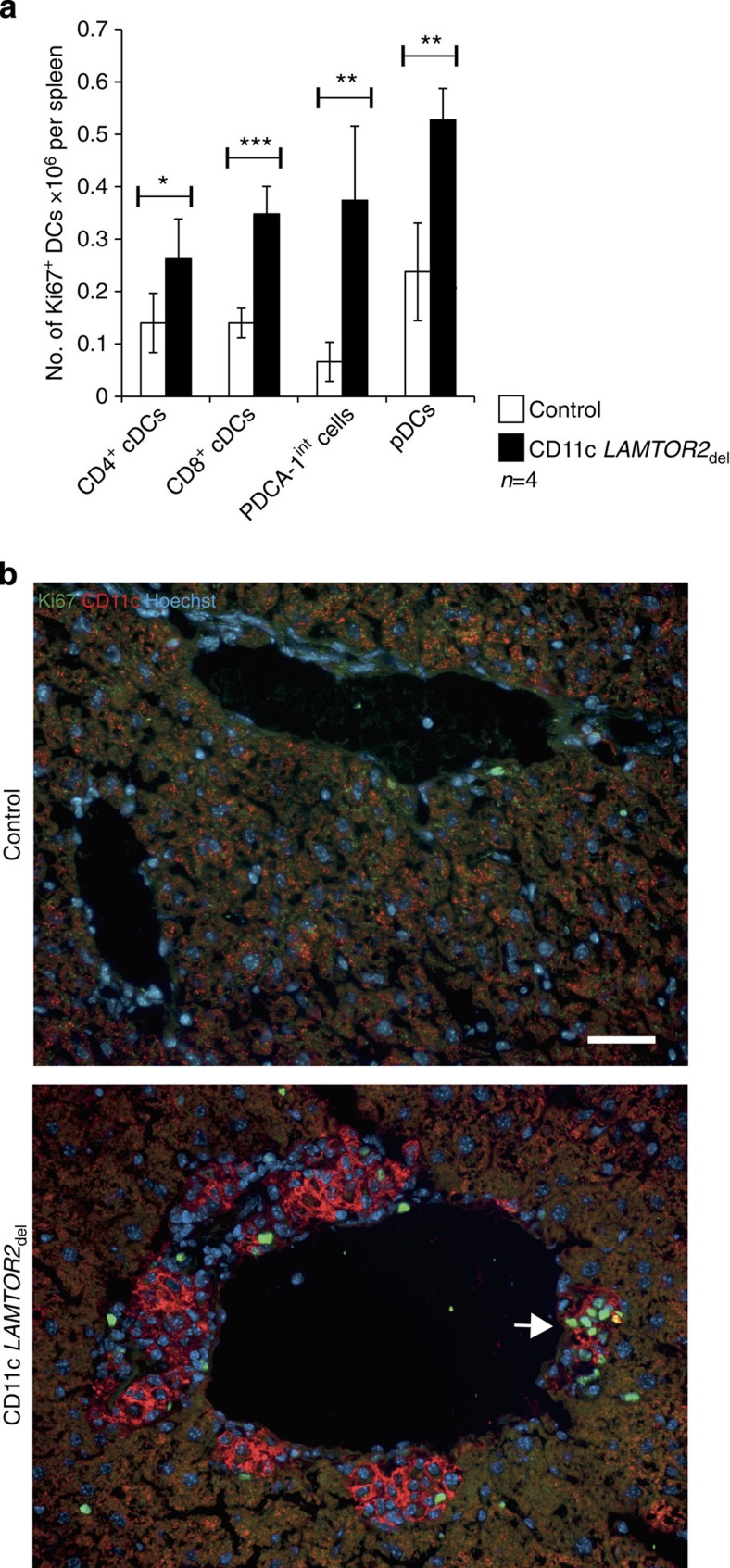
Increased proliferation of DCs. (**a**) Proliferation of the described DC subsets of four control and CD11c *LAMTOR2*_del_ mice was analysed by Ki67 staining. Shown are the numbers of Ki67-positive cells per spleen. Mean±s.d., *P<0.05, **P<0.01, ***P<0,001 as determined by unpaired Student’s *t*-test. (**b**) Frozen liver sections of control and CD11c *LAMTOR2*_del_ mice were stained for CD11c (red), Ki67 (green) and the nucleus (Hoechst). White arrow points at a CD11c-positive infiltrate with Ki67-positive cells. Scale bar, 50 μm.

In a next step, we analysed the proliferation of DCs in the spleen by Ki67 staining of splenic DCs of control and CD11c *LAMTOR2*_del_ mice. All DC populations, including the PDCA-1^int^ one, contained increased numbers of proliferating *LAMTOR2*-deficient DCs (Fig. 5a, Supplementary Fig. 5). In addition, we analysed the infiltrates in the liver for Ki67 expression and found Ki67-positive CD11c+elevated DCs in the infiltrates (Fig. 5b, white arrow).

Taken together, DC-specific deletion of *LAMTOR2* severely affected DC homeostasis resulting in an increase of pre-DCs in the BM and proliferation of cDC, pDCs and CD11c^+^MHCII^+^PDCA-1^int^ cells in the spleen and liver.

### Altered Flt3-receptor trafficking in DCs depleted of *LAMTOR2*

**Figure 6:**
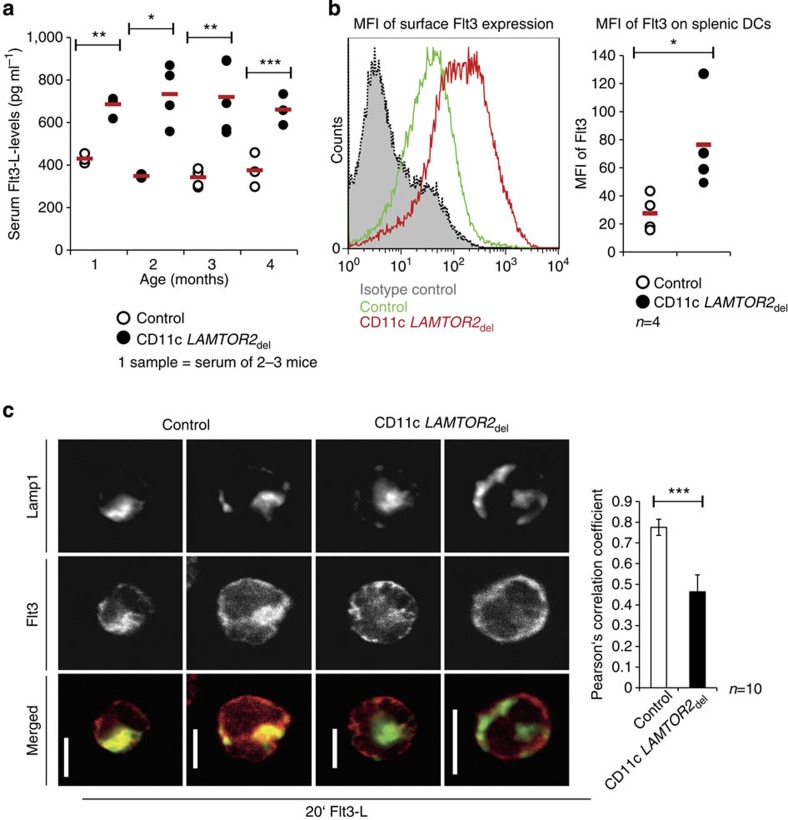
Flt3 is accumulating on the surface of infiltrating DCs. (**a**) Serum samples of control and CD11c *LAMTOR2*_del_ animals starting at the age of 1 month to the age of 4 months were collected and ELISA for Flt3-ligand were performed. Each dot represents the result of a pooled serum sample from three mice. The red bar represents the mean. Mean±s.d., *P<0.05, **P<0.01, ***P<0.001 as determined by unpaired Student’s *t*-test. (**b**) Surface expression of Flt3- receptor on splenic DCs. DCs of control and CD11c *LAMTOR2*_del_ mice were isolated via MACS and stained for CD11c and Flt3. Cells were pre-gated for CD11c and the MFI (mean fluorescence intensity) of Flt3 surface expression was determined as shown in the histogram (green line: control, red line: CD11c *LAMTOR2*_del_). The grey area with the black dotted line corresponds to the isotype control. The graph on the right shows the MFI for Flt3 surface expression of four biological samples. Mean±s.d., *P<0.05 as determined by unpaired Student’s *t*-test. (**c**) A pulse chase experiment stimulating isolated splenic DCs with Flt3-L was performed. DCs of control (left panel) and DC-specific *LAMTOR2*-deficient animals (right panel) were stimulated with 100 ng ml^−1^ Flt3-L for 20 min, fixed and stained for the late endosomal compartment with Lamp1 (green) and for the Flt3-receptor (red). Confocal images were taken. Displayed are two cells each. Scale bar, 5 μm. A graph showing the Pearson Correlation coefficient is depicted (*n*=10 cells). Mean±s.d., ***P<0.001 as determined by unpaired Student’s *t*-test.

The Flt3-ligand (Flt3-L) together with its corresponding receptor Flt3, represents an essential cytokine signal for DC homeostasis, regulating development and differentiation of DC progenitors as well as differentiated DCs^15^,^29^. In mice, the deletion or inhibition of Flt3-L and its receptor leads to a severe reduction of cDCs and pDCs. On the contrary, injection or overexpression of Flt3-L or constitutively active Flt3 signalling leads to the expansion of the DC compartment and subsequently to a myeloproliferative disorder (MPD)^15^,^22^,^29^,^30^,^31^. As not only differentiated DCs but also their pre-DCs express Flt3, we wondered whether the expansion of the DC compartment might be due to an alteration in the Flt3-L availability. The levels of soluble Flt3-L in serum samples of CD11c *LAMTOR2*_del_ mice ranging from 1 to 4 months of age were significantly increased compared with control mice (Fig. 6a). Furthermore, we observed alterations concerning the receptor itself. Surface expression analyses by flow cytometry for Flt3 on isolated splenic DCs (Supplementary Fig. 6a) revealed a significantly increased mean fluorescence intensity (Fig. 6b), indicating an accumulation of the receptor on the surface of DCs of CD11c *LAMTOR*_del_ mice. As it is known that LAMTOR2 plays a crucial part in receptor tyrosine kinase trafficking by controlling endosomal biogenesis (for example, epidermal growth factor (EGF) receptor^10^), we performed pulse chase experiments for Flt3 on isolated splenic DCs. After ligand binding, receptor tyrosine kinases get internalized and are transported through the endocytic pathway to be finally degraded in the lysosome. However, similar to the defect for EGF receptor trafficking seen in *LAMTOR2*-depleted keratinocytes^10^, in which the receptor transfer to the late endosome is delayed, also the Flt3 receptor did not reach the late endosomal compartment. Confocal imaging revealed that 20 min after stimulation with the ligand, the Flt3 receptor was already localizing to late endosomes in control cells. In contrast, no co-localization of Flt3- and Lamp1-positive vesicles could be observed (Fig. 6c).

In summary, we could show that DC-specific deletion of *LAMTOR2* in mice led to increased Flt3-ligand serum levels and an accumulation of the receptor on the plasma membrane because of impaired receptor trafficking towards the late endosome.

### Flt3 surface accumulation leads to increased mTOR activation

**Figure 7:**
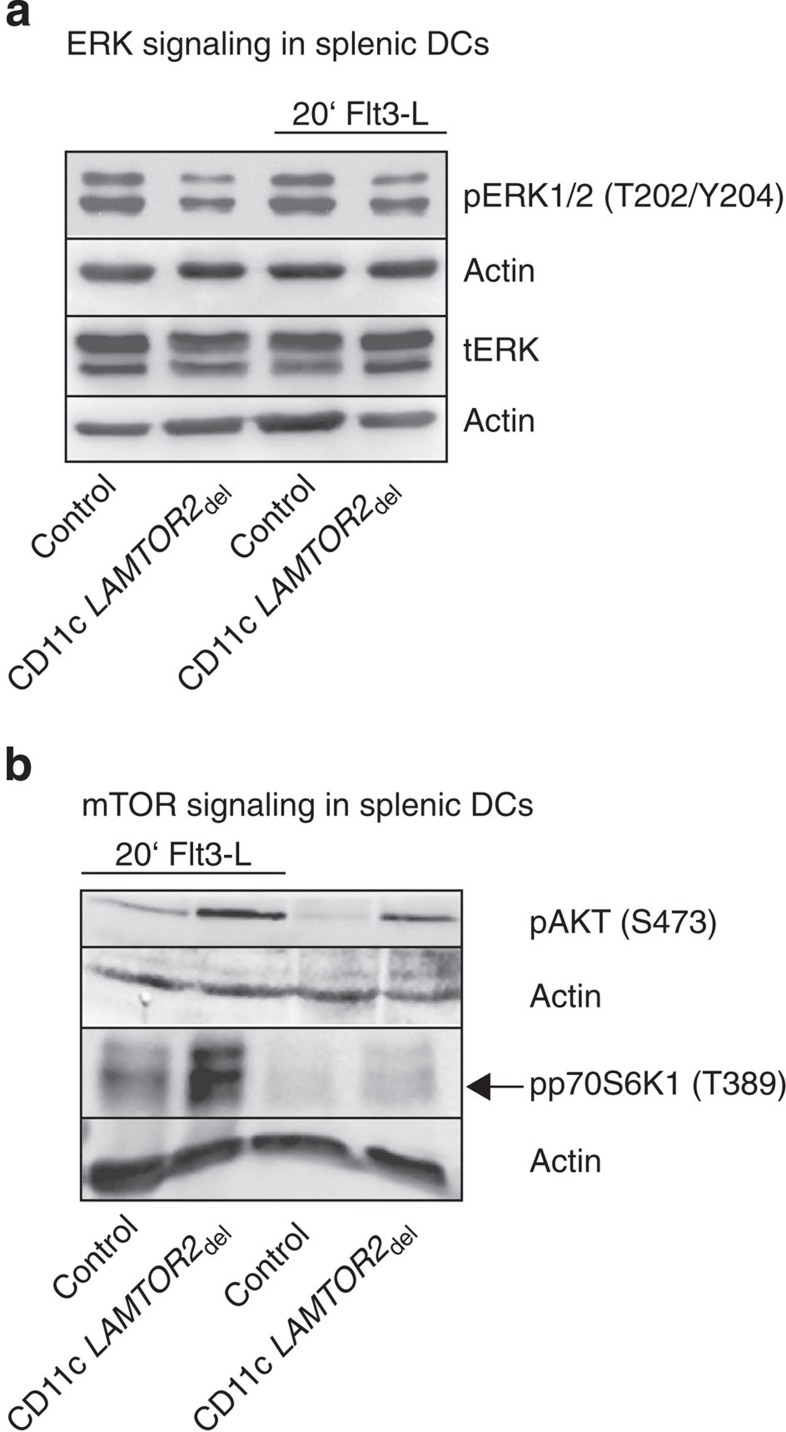
Increased mTOR-signalling in LAMTOR2-deficient splenic DCs. (**a**,**b**) Western blot analysis from lysates of isolated splenic DCs of control and CD11c *LAMTOR2*_del_ mice was performed. One representative out of three biological repetitions is shown. (**a**) Phosphorylation of ERK1 and 2 (Thr202/Tyr204) was determined under steady-state and stimulated (20 min Flt3-L) conditions. Total ERK1/2 was additionally probed and as loading control actin is shown. (**b**) Phosphorylation of AKT (Ser473) and p70 S6K1 (Thr389) was determined under steady-state and stimulated (20 min Flt3-L) conditions. Actin was used as a loading control.

**Figure 8:**
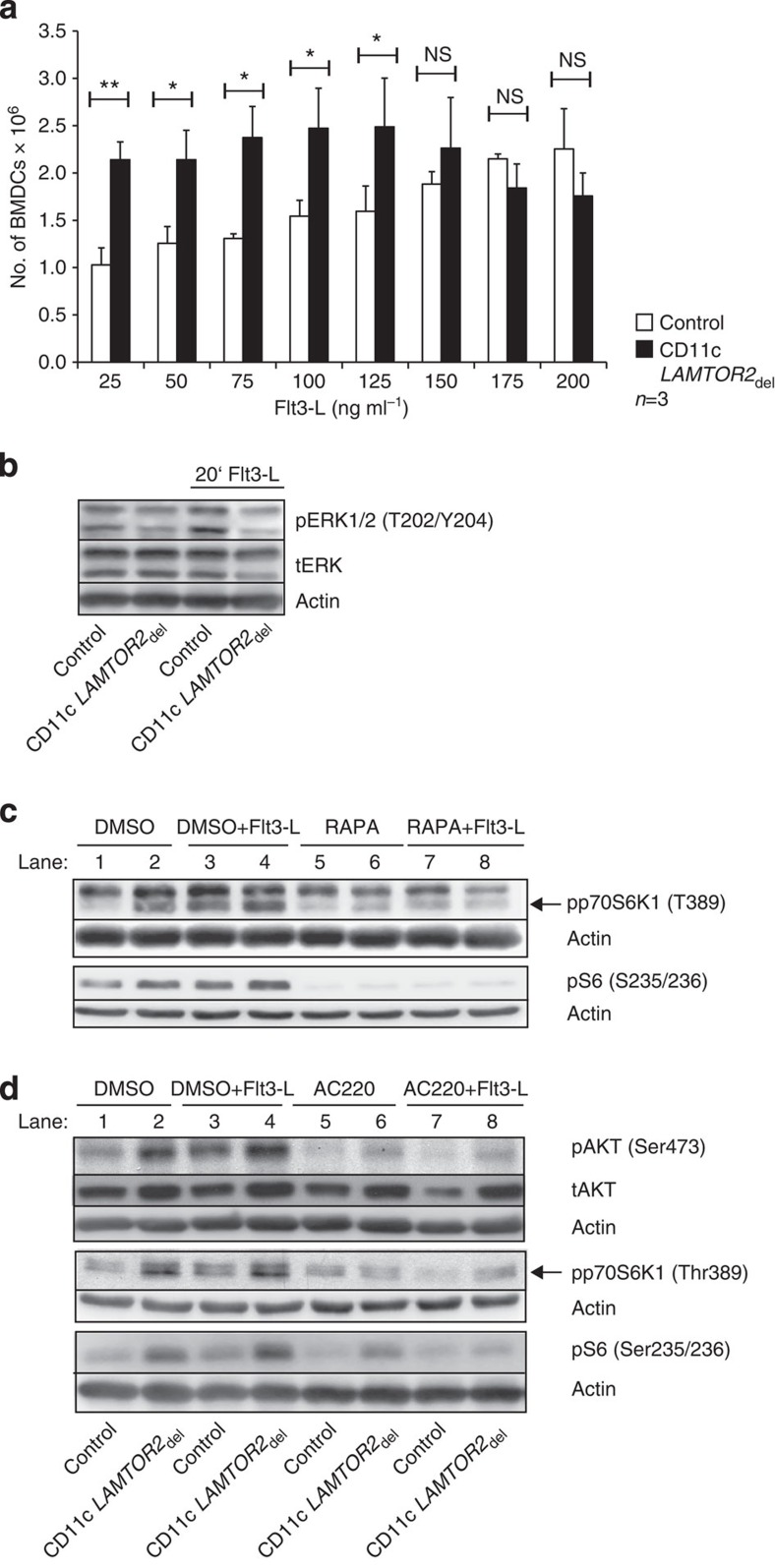
Inhibition of elevated mTOR-signalling in Flt3-L-BMDCs by Rapamycin and AC220. (**a**) BMDCs cultured in the presence of different Flt3-L concentrations (*x* axis) were differentiated for 8 days. 1.5 × 10^6^ BM cells of control and knockout animals were plated per well on day 0. Cell numbers per well on day 8 were determined by counting with a haemocytometer. Presented are three biological repetitions. Mean±s.d., *ns: P>*0.05, *P<0.05, **P<0.01 as determined by unpaired Student’s *t*-test. (**b**) Western blot analysis of BMDCs derived under the influence of Flt3-L was performed and phosphorylation of ERK1/2 was determined on steady state as well after 20 min Flt3-L stimulation. As loading controls, total ERK1/2 and actin were probed. One representative example out of three biological repetitions is shown. (**c**,**d**) Western blot analyses of BMDCs lysates for phospho-AKT (Ser473), phospho-p70 S6K1 (Thr389), phospho-mTOR (Ser2448) and phospho-S6 ribosomal protein (Ser235/236). BMDCs cultured for 8 days in the presence of Flt3-L were pretreated with either Rapamycin (50 ng ml^−1^; **c**) or AC220 (200 ng ml^−1^; **d**), an Flt3-receptor kinase inhibitor, or dimethylsulphoxide (DMSO) as a solvent control for 1 h. Thereafter, cells were briefly (20 min) stimulated with 100 ng ml^−1^ Flt3-L where indicated. As loading controls, tAKT and actin were probed. One representative example out of three biological repetitions is shown.

As it was shown that the activation of PI3K-mTOR signalling, downstream of Flt3, is crucial for DC development and inhibition of this pathway reduces DC numbers^24^,^25^, we decided to investigate the effect of Flt3 receptor accumulation on cell surface in downstream signalling. Previous studies have revealed that loss of LAMTOR2 affects ERK activation as well as mTOR signalling on the late endosome^2^,^3^,^5^. Deletion of *LAMTOR2* in the DC compartment led to a reduction of phosphorylation of ERK in splenic DCs (Fig. 7a). Interestingly, we, however, observed an increased activation of phospho-AKT and phospho-p70 S6 kinase in isolated splenic DC from CD11c *LAMTOR2*_del_ animals as compared with the controls (Fig. 7b). The increased activation of mTOR was observed at steady state as well as after Flt3-L stimulation. For further analysis, we decided to use BM-derived DCs (BMDCs), differentiated in the presence of Flt3-L^32^ (Supplementary Fig. 6b). Titration experiments using different concentrations of Flt3-L revealed that low concentrations of Flt3-L significantly increased numbers of DCs in the BM cultures of the CD11c *LAMTOR2*_del_ mice as compared with the control cultures (Fig. 8a). This result suggests a higher susceptibility of *LAMTOR2-*deficient BMDCs towards Flt3-L. Hence, our *in vitro* BMDC model seems to mimic the DC expansion observed *in vivo* at least partially. In the next step, we analysed the downstream signalling. As expected, BMDC cultures of CD11c *LAMTOR2*_del_ mice showed a decrease in phospho ERK activation (Fig. 8b), however, again an increased activation of AKT and mTOR at steady state and after stimulation with Flt3-L was detected (Fig. 8c,d, lanes 1 to 4). In addition, we added the inhibitors Rapamycin (RAPA; Fig. 8c) and AC220 (Fig. 8d) to the BMDC cultures in order to inhibit mTOR-activation and Flt3 kinase activity, respectively. AC220 (Quizartinib) is a second-generation Flt3 inhibitor developed to treat patients with acute myeloid leukaemia. In approximately 30% of those patients, Flt3 signalling is constitutively active due to mutations in the receptor. It was shown that AC220 has excellent potency, selectivity and pharmacokinetic properties^33^ and is used in clinical trials to treat acute myeloid leukaemia^34^. Both inhibitors caused a decrease in mTOR activation (Fig. 8c,d, lanes 5 to 8) at steady state but, in particular, after Flt3-L stimulation.

In summary, we could show that the increase of plasma membrane-associated Flt3 led to the enhanced activation of the downstream AKT-mTOR signalling pathway in *LAMTOR2*-deficient isolated splenic DCs as well as in BMDCs derived under the influence of Flt3-L. However, this enhanced activation of AKT-mTOR signalling could be abrogated by addition of Rapamycin or AC220. Importantly, the enhanced activation of the Flt3 signalling pathway was further reflected by an increase of DC numbers in *LAMTOR2*-deficient BMDC cultures with limiting concentrations of Flt3-L. This might be an explanation for the DC-related MPD observed *in vivo* in DC-specific *LAMTOR2*-deficient mice.

### Rapamycin reverts the phenotype in CD11c *LAMTOR2*_del_ mice

**Figure 9:**
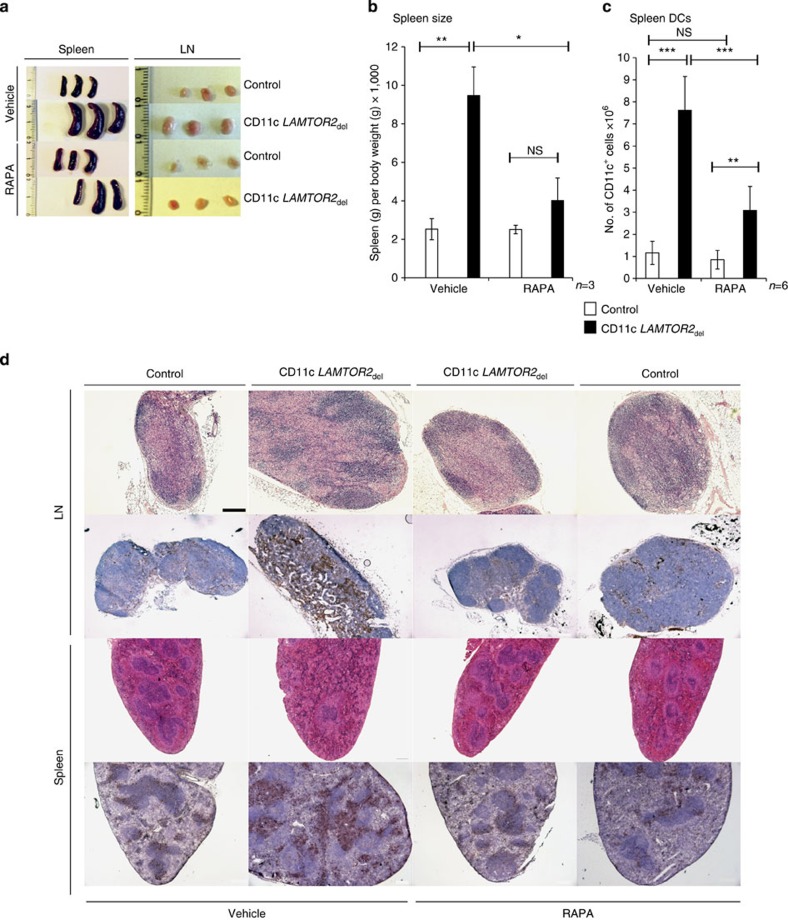
Inhibition of mTOR signalling by Rapamycin *in vivo* restores phenotype. Female mice at the age of 3 months were injected daily for 10 days with 30 μg Rapamycin i.p. and killed on day 10. (**a**) A panel with photographs taken of treated and untreated spleens and LNs from control and deficient animals is shown. Three biological replicates for each genotype and treatment are displayed. (**b**) The graph shows measured spleen weights of control and CD11c *LAMTOR2*_del_ mice treated with Rapamycin and untreated. The spleen weight was correlated to the body weight of the animal. Each group contains three mice. Mean±s.d., *ns: P>*0.05, *P<0.05, **P<0.01 as determined by unpaired Student’s *t*-test. (**c**) Flow cytometry analysis of DC populations of treated and untreated animals were performed. Freshly isolated cells from spleen tissue of control and CD11c *LAMTOR2*_del_ mice were analysed. The total amount of splenic CD11c^+^ cells was calculated (right graph). At least six mice per treatment and genotype are shown. Mean±s.d., **P<0.01, ***P<0.001 as determined by unpaired Student’s *t*-test (**d**) Stained sections of LNs (upper part of the panel) and spleen (lower part) taken from treated and untreated mice from each genotype are shown. Paraffin sections were stained with H&E (first and third row) and cryosections were stained against CD11c (brown) and counterstained with haematoxylin (second and fourth row). Scale bar, 200 μm.

To confirm our hypothesis that increased activation of the AKT-mTOR signalling pathway caused the MPD symptoms in CD11c *LAMTOR2*_del_ mice, we treated the knockout and control animals with 30 μg Rapamycin for 10 days^24^,^25^. By doing so, we could rescue the phenotype of conditional *LAMTOR2*-deficient mice, almost comparable to the one of healthy control mice. Spleen and LN size decreased significantly under the treatment compared with the vehicle-treated CD11c *LAMTOR2*_del_ mice. The size of the organs was almost comparable to that of the control animals (Fig. 9a,b). We investigated the effect of mTOR inhibition on the DC population of the spleen. Therefore, we performed flow cytometry analysis on spleen cell suspensions, where we observed a significant decrease of the DC compartment of CD11c *LAMTOR2*_del_ mice treated with Rapamycin in comparison to the untreated animals. However, we did not observe an effect of Rapamycin treatment in control animals (Fig. 9c). The reduction of DC-related infiltrates in Rapamycin-treated conditional *LAMTOR2-*deficient animals was also observed in histological sections of LNs and spleens. In addition to the regression of DC-infiltrates, we noticed an obvious recovery of the organ architecture of the spleen and LNs in Rapamycin-treated mice (H&E staining, Fig. 9d, first and third row). Finally, immunohistochemistry for the expression of CD11c on frozen organ sections also showed a decrease in infiltrating DCs after mTOR inhibition (Fig. 9d, second and fourth row).

Taken together, inhibition of mTOR signalling by the administration of Rapamycin rescued the phenotype of CD11c *LAMTOR2*_del_ mice and restored the size of various organs by suppressing the expansion of DCs.

### Flt3 inhibition by AC220 treats MPD in CD11c *LAMTOR2*_del_ mice

**Figure 10:**
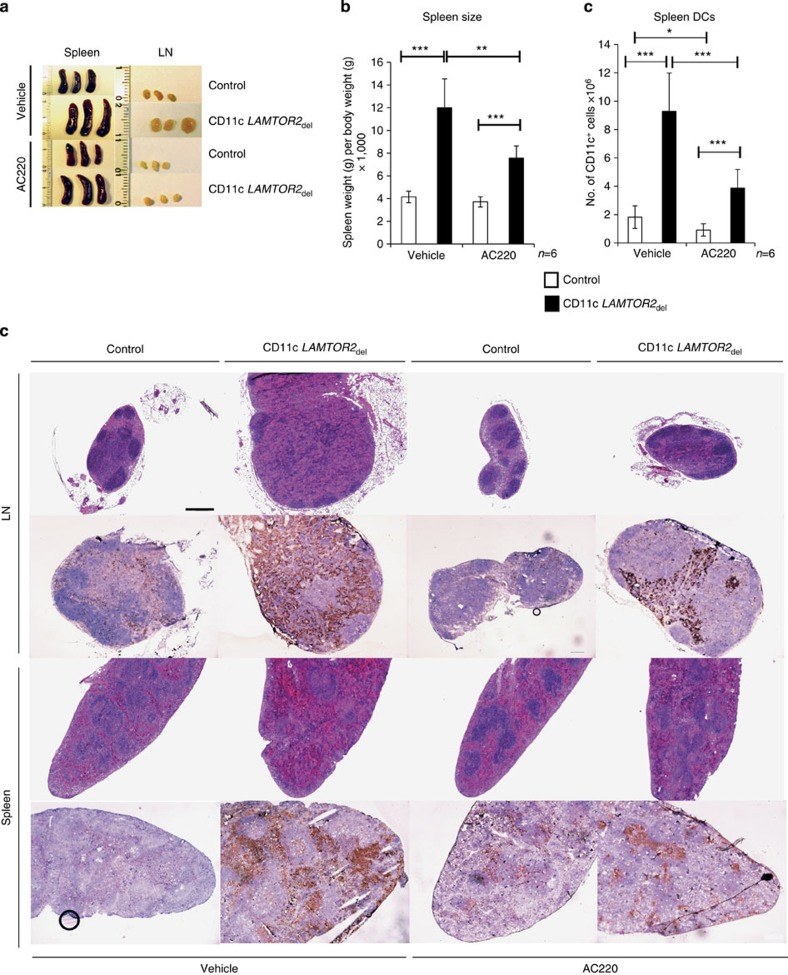
Inhibition of the Flt3-kinase receptor by AC220 induces phenotypic regression. AC220 (10 mg kg^−1^) was administered orally for 28 days by gavage. Mice were age and sex matched (2.5 months). (**a**) Spleens and LNs from treated and untreated control and CD11c *LAMTOR2*_del_ are shown. (**b**) The spleen weight of treated and untreated animals of each genotype (six mice) was calculated in correlation to their body weight (as indicated on the *y* axis). Mean±s.d., *P<0.05, **P<0.01, ***P<0.001 as determined by unpaired Student’s *t*-test. (**c**) Flow cytometry analysis of DC populations of treated and untreated animals. Spleen tissue from control and CD11c *LAMTOR2*_del_ mice was analysed for CD11c^+^ cells. At least six biological replicates per treatment and genotype are displayed. Mean±s.d., *P<0.05, ***P<0.001 as determined by unpaired Student’s *t*-test. (**d**) Stained sections of LNs (upper part of the panel) and spleen (lower part) taken from treated and untreated mice from each genotype are shown. Paraffin sections were stained with H&E (first and third row) and cryosections were stained against CD11c (brown) and counterstained with haematoxylin (second and fourth row). Scale bar, 200 μm.

In a second approach, we aimed to suppress DC expansion in the CD11c *LAMTOR2*_del_ mice by directly blocking Flt3 kinase activity. In another mouse model developing MPD due to enhanced wild-type Flt3 signalling, AC220 could hold the disease in remission during the therapy period^35^. The mice were treated daily for 28 days with 10 mg kg^−1^ AC220 or vehicle by oral gavage and then killed to investigate the effect on the DC compartment. AC220-treated conditional *LAMTOR2*-deficient mice revealed a significant reduction in size of spleen and LNs as compared with untreated mice. Also a decrease of the LN size, almost back to control size, could be observed (Fig. 10a–c). Flow cytometry analyses of spleen suspension cells from those animals marked a significant drop of the DC numbers. Furthermore, analysis of spleen and LN morphology by H&E staining (Fig. 10d, first and third row) was performed. Both organs of CD11c *LAMTOR2*_del_ mice displayed a partial restoration of the distinct lymphocytic compartments upon treatment, which was more prominent in the LN. Moreover, immunohistochemistry revealed less infiltrating DCs (Fig. 10d, second and fourth row).

Thus, treatment of the CD11c *LAMTOR2*_del_ mice with a specific Flt3 inhibitor, AC220, led to the remission of the MPD symptoms. DC expansion in the spleen and LNs was reduced, leading to a normalization of organ morphology and size.

## Discussion

LAMTOR2 plays a critical role in tissue homeostasis and immunity^10^,^11^,^12^,^27^. Here, we contribute insights into the function of LAMTOR2, as part of the Ragulator/LAMTOR complex, which operates as molecular switch on the late endosome to differentially regulate mTOR and MAPK-signalling downstream of the Flt3-receptor. As a consequence of DC-specific deletion of *LAMTOR2*, a disruption of the entire complex is observed, followed by a deregulation of Flt3 sorting and signalling. This severely affects DC homeostasis and culminates in a MPD.

LAMTOR2 was shown to play a crucial role for innate and adaptive immunity, as described in patients harbouring a hypomorph *LAMTOR2* allele and as consequence suffering from recurrent broncho-pulmonary infections. One explanation for their immunodeficiency syndrome are defects in their endo- and lysosomal biogenesis resulting in insufficient digestion of bacteria by neutrophiles^11^. Along those lines go observations that mice with *LAMTOR2*-deficient macrophages cannot properly fight *Salmonella* infections due to defects in their phagosomal compartment^12^. Recent findings strengthened the assumption that LAMTOR2 also plays a crucial role in DCs as major inducers of the adaptive immunity. Preceding investigations of the same conditional CD11c *LAMTOR2*_del_ mouse model revealed a permanent loss of epidermal Langerhans cells, a subpopulation of skin DCs, shortly after birth because of reduced proliferation and increased apoptosis. Further investigations of LN resident DC populations showed no alterations at the age of 6–8 weeks^27^. The massive expansion of cDCs and pDCs we describe here is observed only after 10–12 weeks, suggesting that the myeloproliferative syndrome develops later on in ageing mice. Interestingly, unlike Langerhans cells, cDC and pDCs depend strongly on Flt3 receptor signalling through the AKT/mTORC1 pathway for their differentiation and development^36^. Hackstein and colleagues observed that the *in vivo* administration of Rapamycin, a classical inhibitor of mTOR, inhibits the effect of Flt3-L as an important DC growth factor^25^. Also, the *in vitro* DC development of pDCs and steady-state DCs is impaired after Rapamycin treatment and conversely, deletion of the PI3K inhibitor PTEN, facilitates Flt3-L-induced DC expansion^24^.

mTOR signalling is an essential pathway to promote cell growth and proliferation under nutrient-rich conditions by, for example, protein synthesis and repression of autophagy via amino-acid sensing. Amino acid-induced mTORC1 activation takes place at the membrane of late endosomes. The Ragulator/LAMTOR complex, including LAMTOR2, was shown to be essential for recruiting the Rag proteins to the lysosome, where those, in response to amino-acid stimuli, interact with and activate mTORC1 (ref. 3). In contrast to the intrinsic amino acid-dependent mTOR activation, extrinsic stimuli like cytokines and growth factors induce mTOR signalling via receptor tyrosine kinases. This is followed by the phosphorylation of the tuberous sclerosis complex, which then releases its inhibiting effect on Ras homolog enriched in brain (RHEB), the activator of mTORC1 (ref. 37).

As the LAMTOR complex is crucial for the activation of MEK/ERK and mTORC1 on late endosomes and deletion of *LAMTOR2* destabilizes the so far known components, we investigated how both signalling pathways would be affected. As expected, we observed differences of the activation state of ERK1/2 in isolated splenic DCs as well as BMDCs derived under the influence of Flt3-L. Upon deletion of *LAMTOR2,* ERK activation was reduced as was also shown previously for *LAMTOR1* (ref. 4), *LAMTOR2* (ref. 10) and LAMTOR3 (ref. 38) depletion. Interestingly, stimulation with Flt3-L did not increase the activation of ERK, confirming a distinct Flt3 downstream signalling pathway inducing the MPD.

Next, we analysed the AKT/mTOR pathway in DCs of CD11c *LAMTOR2*_del_ mice. Unexpectedly, we observed there a hyper-activation of AKT, p70 S6K1 and S6 protein in splenic DCs and Flt3-L-BMDCs depleted of *LAMTOR2*. This could be measured under steady state as well as under stimulation with Flt3-L. Thus, we assume that extrinsic stimulation of mTORC1 via the Flt3 receptor must bypass LAMTOR complex-dependent mTORC1 signalling on endosomes and most likely takes place at a different subcellular localization, for example, as recently discussed for the cytosolic compartment^39^.

The consequences of such uncoupled signalling, involving two of the major signalling pathways regulating cell growth and proliferation, are severe. Although ERK signalling is abolished, the elevated mTOR signalling induces a massive expansion of pre-DCs and DCs resulting in a MPD. As a possible mechanism, we could identify a transport defect of the Flt3 receptor to the lysosome followed by an increase of the receptor on the surface of DCs. This finding underlines the role of LAMTOR2 in receptor trafficking and endosomal biogenesis, as it goes in line with previous results, showing slower transport of the EGF-receptor in LAMTOR2-depleted mouse embryonic fibroblasts (MEF) as well as in keratinocytes to the lysosome^10^.

In addition, we also measured increased levels of Flt3-L in the serum of CD11c *LAMTOR2*_del_ mice. Flt3-L is a crucial cytokine during haematopoiesis and elevated Flt3-L levels, through *in vivo* administration, not only affect stem cells and myeloid progenitors but also lead to an elevation of immature B cell, NK cell and DC numbers in lymphoid organs and can finally result in a MPD^40^,^41^. Although most cell types express a certain amount of Flt3-L mRNA, stromal cells and activated, proliferating T cells are the major producers^15^,^42^,^43^. As we observed an increased expression of maturation markers on splenic DCs in the CD11c *LAMTOR2*_del_ mice, we speculate that this might be also the result of a failure in LAMTOR2-dependent receptor sorting and consequently as shown, T-cell activation takes place that by itself might lead to elevated Flt3-L secretion. Another possibility could be a reduced consumption of ligand by DCs. This might be explained by the stuck receptor on the cell surface. The bound ligand would not be disassociated and a constitutive active receptor signalling, as observed, would be the consequence.

Interestingly, the observed increased Flt3-L levels did neither affect MDP and CDP numbers in the BM nor numbers of immature B cells and NK cells in the spleen. However, a significant elevation of CD11c^+^ pre-DCs in the BM and increased proliferation of DCs in the spleen and liver were assessed. This observation led to the conclusion that not the elevated ligand levels alone but their combination with the increased presence of Flt3 on the cell surface causes the pronounced expansion of DCs as well as CD11c^+^ pre-DCs. The impact of this scenario leads to the above-described paradox in the uncoupled Flt3 receptor downstream signalling.

To further underline our finding, we confirmed that *in vitro* as well as *in vivo* inhibition of Flt3-receptor-induced mTOR activation can be achieved by Rapamycin and the second-generation Flt3-receptor kinase inhibitor AC220 and that this leads to a remission of the MPD symptoms in CD11c *LAMTOR2*_del_ animals. Furthermore, we observed in AC220-treated control animals already a significant reduction of DC numbers in the spleen emphasizing the specific effect of the Flt3-inhibitor on DC development. However, treatment of control animals with the allosteric mTOR inhibitor Rapamycin did not show a DC reduction. Here we speculate that other signalling pathways downstream of the Flt3 receptor, for example, STAT3 (ref. 44), might compensate for mTORC1 inhibition.

This leads to the conclusion that LAMTOR-complex-mediated mTOR signalling quality might respond differently to intrinsic, amino acid-induced, versus extrinsic, Flt3-L-induced, stimuli. Therefore, our results support a model in which a molecular switch triggers mTOR activation but ERK downregulation dependent on growth factor, Flt3-ligand, signalling in the absence of LAMTOR2.

## Methods

### Mice

CD11c Cre mice^26^ were provided by B. Reizis (Columbia University, New York, NY, USA). The *LAMTOR2*_f/f_ mice^10^ were generated in our laboratory. Both mouse strains were backcrossed onto a C57BL/6 background for at least ten generations. To obtain CD11c Cre *LAMTOR2*_del/+_ and CD11c Cre *LAMTOR2*_del/del_ mice, the CD11c Cre mice were crossed with the *LAMTOR2*_f/f_ mice. The CD11c Cre was kept hemizygous. Genomic DNA was isolated from ear biopsies with Viagen DirectPCR DNA Extraction System overnight and the genotype was determined by PCR^10^,^26^. If not stated differently in the figure legend, sex-matched mice at the age of 3 months were used for the experiments. No randomization strategy was used.

### *In vivo* experiments

Animal experiments were performed in accordance with the Austrian legislation (TVG BGBl no. 501/1989, i.d.F. BGBl I no. 162/2005) and were granted by the Austrian Federal Ministry of Science and Research (code: 66011/205-II/3b/2011).

*Rapamycin*. Mice at the age of 3 months were injected i.p. for 10 days with 30 μg Rapamycin (LC Laboratories) in PBS with 5% dimethylsulphoxide and 10% ethanol or vehicle alone^24^,^25^.

*AC220 (Quizartinib)*. AC220 (LC Laboratories) at 10 mg kg^−1^ was administered to female mice (10 weeks old) daily for 4 weeks by gavage. AC220 was reconstituted in 22% hydroxypropyl-β-cyclodextrin (Sigma-Aldrich). A corresponding control group was given only the vehicle^33^.

For both experiments, animals were killed 24 h after the last treatment and organs were analysed by flow cytometry and histology.

*BMDCs*. BMDCs were generated in the presence of Flt3-L but in the absence of GM-CSF, as described before^32^. In brief, BM cells were isolated and after erythrocyte lysis, 1.5 × 10^6^ cells were plated per well in a 24-well plate in IMDM (Gibco) supplemented with 10% FCS (Lonza) and 50 nM mercaptoethanol. 200 ng ml^−1^ Flt3-ligand (Shenandoah Laboratories) was added, and at day 8, the DCs in the supernatant were harvested. Purity was determined by flow cytometry analyses.

*Splenic DCs*. Cells were isolated by MACS Cell Separation (Miltenyi Biotec) according to the manufacturer’s protocol. For indicated experiments either the CD11c or the pan-DC microbeads were used. Purity was controlled by fluorescence-activated cell sorting (FACS) analysis.

*T cells, B cells, pDCs and PDCA-1^int^ cells*. Cells were analysed and sorted by a BD Bioscience FACS-Canto or Aria (BD Bioscience).

### Histology, immunohistochemistry and immunofluorescence

For histological analyses, organs were fixed in Bouin’s solution and embedded in paraffin. Sections of 5 μm were cut and stained with H&E. For immunohistochemistry and immunofluorescence, organs were embedded in Tissue-Tek O.C.T. compound, 5 μm sections were cut and fixed for 8 min in 100% acetone at −20 °C. For immunofluorescence, sections were blocked for 30 min in 5% goat serum in PBS and incubated for 1 h with the following antibodies: CD11c (1:100, clone N418, eBioscience), PDCA-1 (1:100, clone 120G8.04, Dendritics) and Ki67 (1:200, clone K-2, Novacastra), followed by a 30 min incubation with the secondary antibody (Alexa Fluor 488 goat anti-rat, Alexa Fluor 568 goat anti-hamster, 1:1,000, Invitrogen) at room temperature. Sections were embedded in Mowiol (Sigma-Aldrich). For immunohistochemistry of CD11c, fixed sections were blocked for 5 min with 0.03% H_2_O_2_ and 0.3% goat serum in PBS, followed by a blocking with the Avidin-Biotin blocking kit (VectorLabs). Sections were then incubated for 1 h with the CD11c antibody (eBioscience), washed and incubated for 30 min with the biotinylated goat anti-hamster secondary antibody (VectorLabs). After washing, sections were incubated for 5 min in Vectastain ABC reagent (VectorLabs), followed by incubation with AEC (3-amino-9-ethylcarbazole), the peroxidase substrate (VectorLabs). Pictures were taken on a Zeiss Axio Imager M.1 equipped with a CoolSnapHQ2 CCD camera (Photometrics) or an AxioCam HRc (Carl Zeiss MicroImaging, Inc.). The acquisition software AxioVs40V4.5.0.0 (Carl Zeiss MicroImaging, Inc.) or VisiView (Visitron) were used.

### Pulse chase experiments

Splenic DCs were pre-cooled to 4 °C on Superfrost slides (Menzel) and incubated for 10 min with 100 ng ml^−1^ Flt3-ligand. Cells were then washed with ice-cold medium and incubated for 20 min at 37 °C, immediately fixed for 1–2 min in 4% formaldehyde (FA) and blocked for 30 min in 5% goat serum and 0.05% Saponin (Sigma-Aldrich) in PBS. Cells were then incubated for 2 h with antibodies for Flt3 (1:100, H-300, Santa Cruz) and Lamp1 (1:200, 1D4B, Pharmingen) in blocking buffer, washed and incubated for 30 min with the secondary antibodies (Alexa Fluor 488 goat anti-rat, Alexa Fluor 568 goat anti-hamster, 1:1,000, Invitrogen) at room temperature. Confocal pictures were taken at a confocal laser-scanning microscope (LSM510 Meta; Carl Zeiss MicroImaging, Inc.) with a × 63 plan-Apochromat NA 1.4 oil objective (Carl Zeiss MicroImaging, Inc.). For the acquisition of images, LSM Image Examiner software (version 3.1.0.117; Carl Zeiss MicroImaging, Inc.) was used.

### ELISA

Blood samples were collected and centrifuged for 20 min for 1,500 r.p.m. after coagulation. Serum was stored at −80 °C. Mouse Flt3- ligand Quantakine ELISA was performed according to the manufacturer’s assay procedure (R&D Systems).

### Multiplexing immunoassays

Serum was retrieved as described above. Spleen cell supernatants were prepared from 5 × 10^6^ spleen cells, which were cultured overnight in R10 medium. For the stimulation assays with CpG, splenic DCs were isolated with the pan-DC microbeads (Milteny) and 5 × 10^5^ cells were plated in R10 medium containing 20 ng ml^−1^ GM-CSF and 2 μg ml^−1^ GpG (1,668, TIB Molbiol). The next day, the culture supernatants were collected. The multiplexing immunoassays ProcartaPlex Mouse Th1/Th2/Th9/Th17/Th22/Treg Cytokine Panel (17 plex), with added Simplex IL-1α and the 2-plex IFN-α and IFN-β, were purchased from eBioscience and measured in a FlexMAP3D instrument.

### Flow cytometry analyses

Flow cytometry analyses were performed on BMDCs, BM, spleen and LN single-cell suspensions cells. Cell suspension of the spleen and LNs were prepared by cutting the tissue into small pieces and digestion for 25 min at 37 °C, in a shaking waterbath with 0.5 mg ml^−1^ Collagenase D (Roche) and 120 μg ml^−1^ DNAse (Merck) in Hanks buffered salt solution (PAA). After adding 200 μl of 0.5 M EDTA, pH 8 (AppliChem), cell suspension was pressed through a 70-μm cell strainer, washed and erythrocyte lysis was carried out. For FACS analysis, cells were washed with 1% BSA in PBS, blocked for 20 min with FcγRIII/II- block (1:10,000, clone 2.4G2, BD Bioscience) and then incubated for 1 h with the specific fluorophore-conjugated antibodies (APC-CD11c (1:400, clone N418), APC-CD8a (1:400, clone 53-6.7), APC-CD4 (1:400, clone RM4-5), PE-CD8a (1:200, clone 53-6.7), FITC-Gr-1 (1:100, clone RB6-8C5), PE-c-Kit (1:100, clone 2B8), FITC-CD3e (1:100, clone 145-2C11), PE-IgM (1:100, clone r6-60.2), FITC-IgD (1:100, clone 11-26c.2a), CD40-PE (1:100, clone 323), CD44-APC (1:100, clone IM7) from BD Bioscience; PE-CD11b (1:100, clone M1/70), PE-Cy7-CD115 (1:200, clone A2F10), APC-Flt3 (1:100, clone A2F10), PerCP-Cy5.5-MHCII (1:100, clone M5), PE-CD11c (1:100, Clone N418), Brilliant Violett 510-CD3 (1:100, clone 17A2), PE-Cy7-NKp46 (1:100, clone 29A1.4), Brilliant Violett 510-CD4 (1:100, clone RM4-5), Brilliant Violett 421-CD8a (1:100, clone 53-6.7), APC-CD86 (1:100, clone GL1), PE-Cy7-CD62L (1:100, clone MEL-14), PerCPCy5.5- CD69 (1:100, clone H1.2F3), PE-PD-1 (1:100, clone MH5A), APC-PD-L1 (1:100, clone 10F.9G2) from BioLegend; FITC-PDCA-1 (1:100, clone 927), PE-CD4 (1:100, clone GK1.5), CD19-PerCP-Cy5.5 (1:100, clone 6D5), FITC-lineage Mix from eBioscience) and respective isotype controls. For Ki67-PE (1:200, clone 16.A8, BioLegend) staining, surface markers were stained as described before. Then cells were fixed, permeabilized and Ki67-stained with the Foxp3/Transcription Factor Fixation/Permeabilization Concentrate and Diluent Solution kit (eBioscience) according to the manufacturer’s protocol. Cells were analysed by a Calibur (BD Bioscience) flow cytometer and data analysis was performed with FlowJo software.

### Western blot analyses

For phosphorylation analysis of AKT, mTOR, p70 S6 Kinase 1, S6 ribosomal protein and Erk1/2, cells were stimulated for 20 min with Flt3 ligand. In addition, where stated, cells were treated with Rapamycin (50 ng ml^−1^, LC Laboratories) or AC220 (100 ng ml^−1^, LC Laboratories) for 1 h, before stimulation. Cell lysates of BMDCs and splenic DCs were prepared^2^ and separated on a SDS–polyacrylamide gel electrophoresis. Membranes were probed after blotting and blocking against primary antibodies (LAMTOR2, LAMTOR3 (ref. 10), pAKT (1:500, clone D9E), AKT (1:500–1,000), pp70S6K1 (1:1,000, clone 1A5), pERK1/2 (1:500, clone E10), ERK1/2 (1:1,000, clone 137F5), pS6 (1:1,000, clone D57.2.2E) from Cell Signaling; Actin (1:5,000, clone C4) from Millipore; LAMTOR1 (1:1,000), LAMTOR4 (1:1,000) from Atlas; LAMTOR5 (1:500, clone C16) from Santa Cruz) overnight at 4 °C in blocking buffer (3% BSA, 1 mM EDTA, pH8, 0.05% Tween 20, 6 mM Sodium Azide, pH 5.2). Membranes were washed with Tris -buffered saline (50mM Tris-HCl, pH 7.6; 150mM NaCl) with 0.05% Tween 20 and incubated with the secondary antibodies (anti-mouse-/anti-rabbit IgG peroxidase antibody, 1:5,000 (Sigma)). Detection was performed by chemiluminescence. Uncropped western blots are provided in Supplementary Figs 7 and 8.

### Real-time quantitative PCR

RNA of Flt3-L-BMDCs, sorted B and T cells was extracted with a ‘High Pure RNA isolation kit’ (Roche) according to the manufacturer’s manual. RNA samples were subjected to reverse transcription analysis using Revert Aid First Strand cDNA Synthesis Kit (Thermo Fisher Scientific) with oligo dT primers. Quantitative PCR analysis was performed using the DyNAmo Flash SYBR Green qPCR kit (Thermo Fisher Scientific). Following *LAMTOR2* primers were used: forward: 5′-TCTGGGCCGCGTATGATAGG-3′ and reverse: 5′-CACGCTGTTATGATGCTGCTACTT-3′. Results were normalized to *GAPDH* cDNA. The amplification of cDNA was performed by PIKOREAL96 (Thermo Fisher Scientific). Resulting data were analysed via two-tailed type 2 Student’s *t*-test.

## Author contributions

J.M.S., F.S, C.H.T., C.H., A.H. and J.B. did *in vitro* and *in vivo* experiments and analysed data. N.R and P.S. contributed with experimental design and edited the manuscript. L.A.H. conceived and supervised the study, and wrote and edited the manuscript.

## Additional information

**How to cite this article:** Scheffler, J. M. *et al.* LAMTOR2 regulates dendritic cell homeostasis through FLT3-dependent mTOR signalling. *Nat. Commun.* 5:5138 doi: 10.1038/ncomms6138 (2014).

## Supplementary Material

Supplementary Figures Supplementary Figures 1-8
