# Supplementary material for: LAMTOR2 regulates dendritic cell homeostasis through FLT3-dependent mTOR signalling
Source: Nat Commun. 2014 Oct 22;5:5138. doi: 10.1038/ncomms6138 (PMC4220488; doi:10.1038/ncomms6138)
Supplement: Supplementary Figures — 1-8 [file ncomms6138-s1.pdf]

Fig. S1

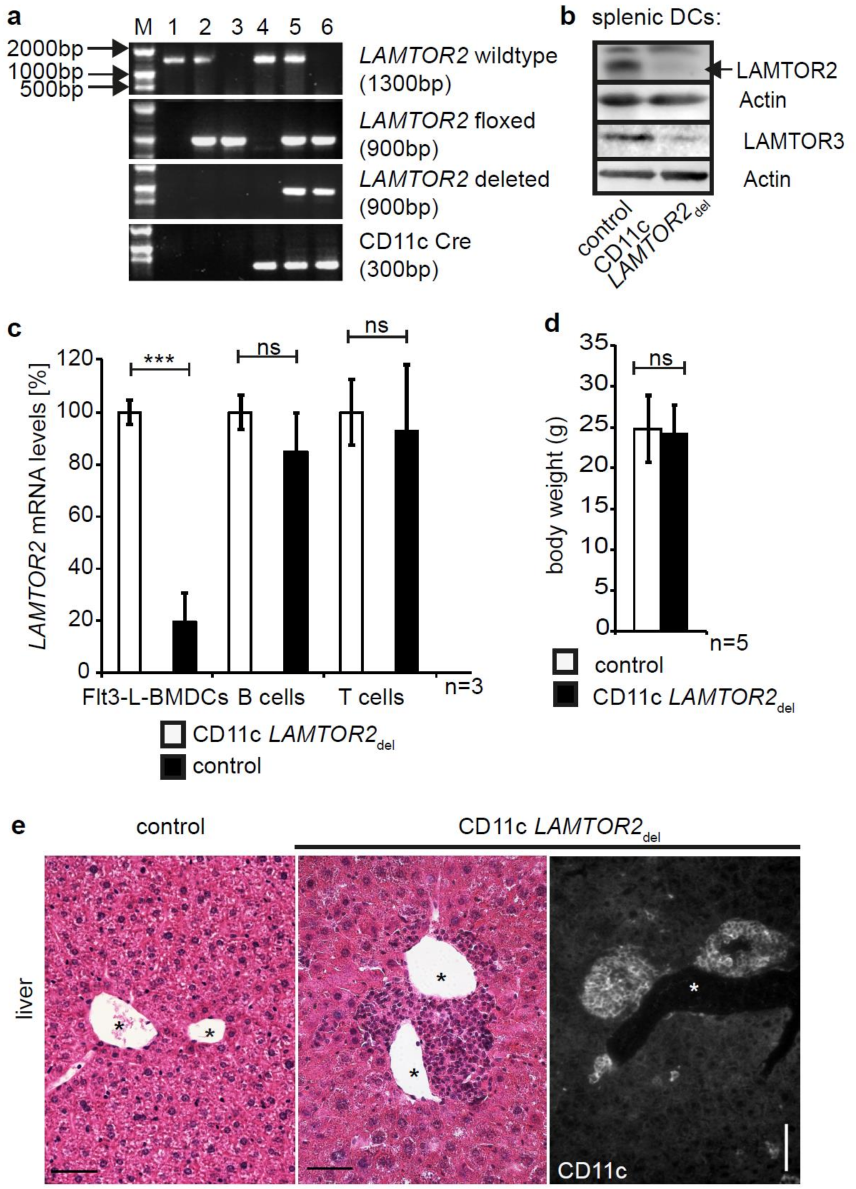

Supplementary Figure 1:

(**a**) Genotype results from murine ear samples taken 3 weeks after birth are shown. PCR primers to demonstrate CD11cCre (300bp), to distinguish wildtype (1300bp) from floxed (900bp) and deleted (900bp) *LAMTOR2* alleles were used to identify all possible genotypes. M: DNA-ladder, 1: *LAMTOR2*$_{+/+}$, 2: *LAMTOR2*$_{f/+}$, 3: *LAMTOR2*$_{f/f}$, 4: CD11c *LAMTOR2*$_{+/+}$, 5: CD11c *LAMTOR2*$_{del/+}$, 6: CD11c *LAMTOR2*$_{del/del}$. (**b**) Western blot of splenic DC lysates from control and CD11c *LAMTOR*$_{del}$ mice was performed against LAMTOR2 and 3. Actin was used as loading control. (**c**) mRNA levels of LAMTOR2 in FL-BMDCs, B cells and T cells were measured. Mean±SD, *ns p>0.05, *** p<0.001* as determined by unpaired Student's t-test. (**d**) Body weight of control and CD11c *LAMTOR2*$_{del}$ mice (n=7 per genotype), Mean±SD, *p=n.s.,* as determined by unpaired Student's t-test. (**e**) Paraffin sections of 3 months old control and CD11c *LAMTOR2*$_{del}$ mice for liver were stained with H&E. Cryosections were stained for CD11c. "*" indicates the sinus. Scale bar = 50µm.

Fig. S2

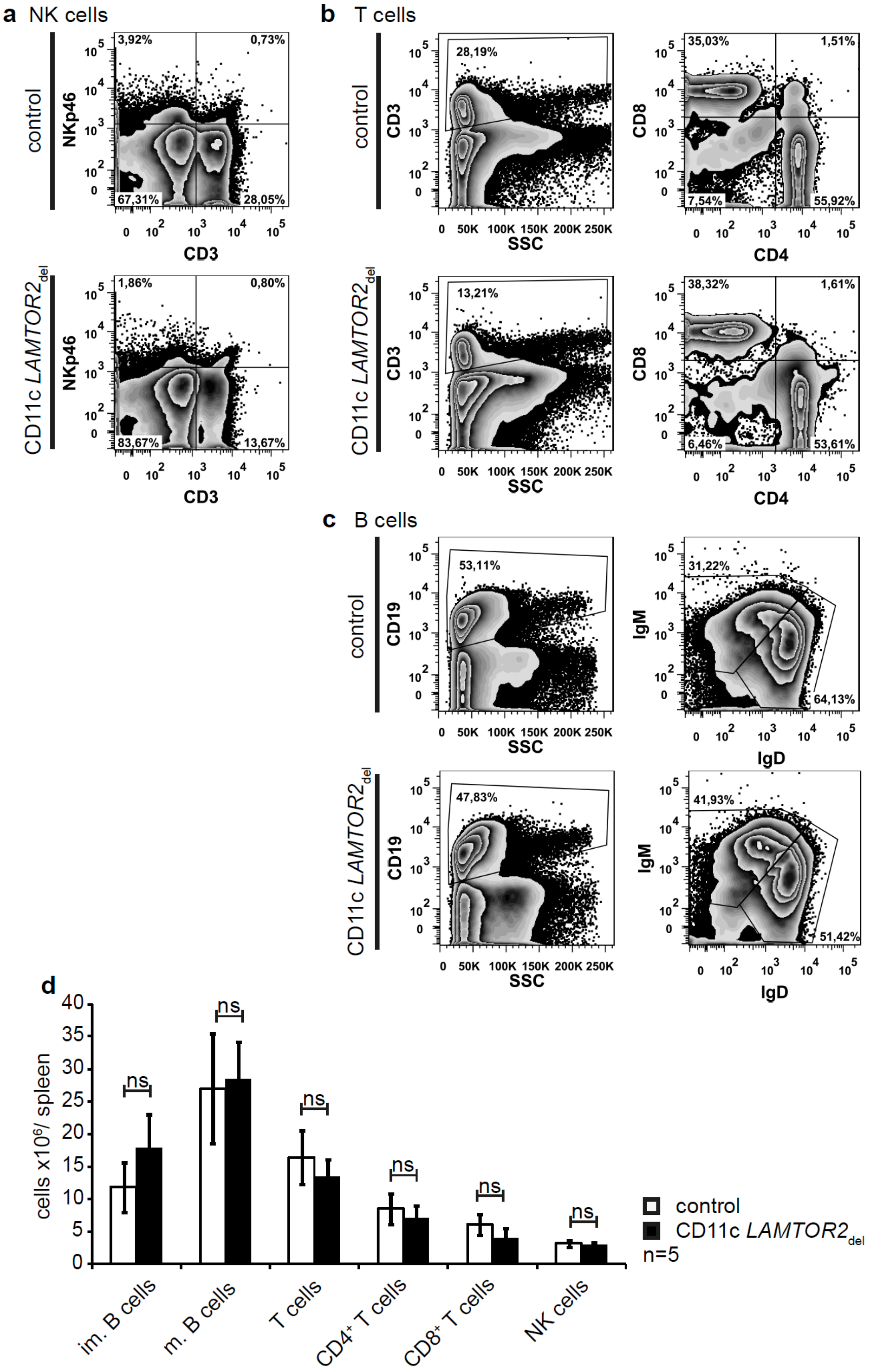

Supplementary Figure 2:

(**a, b, c, d**) Analyses of major spleen cell populations by flow cytometry. One representative for each genotype is shown. (**d**) Combined analyses of 5 individual measurements for each genotype are shown. Mice at the age of 3 months were analyzed. Mean±SD, *ns p>0.05* as determined by unpaired Student's t-test.

Fig. S3

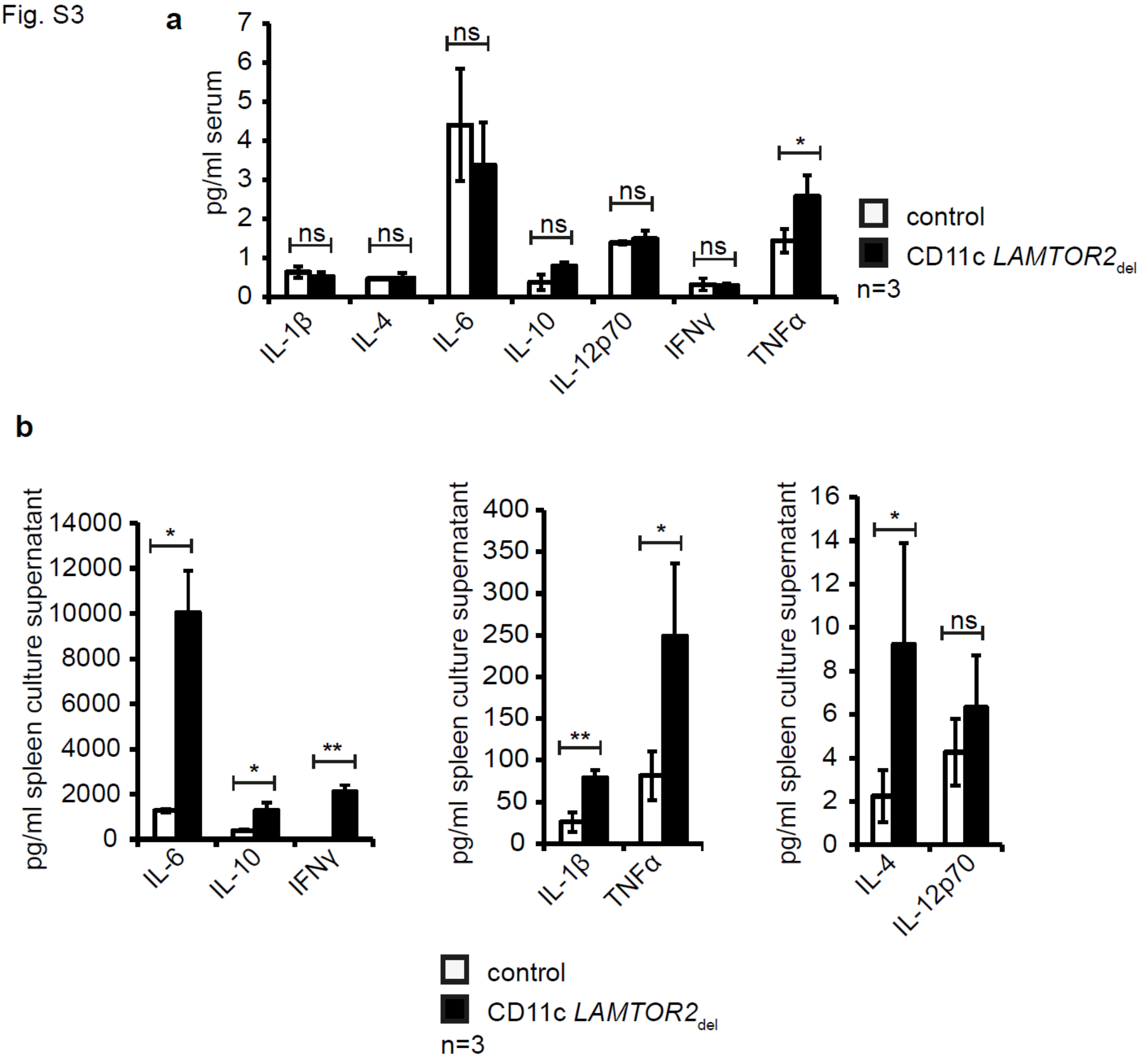

Supplementary Figure 3:

(**a**) Serum samples of 3 control and CD11c *LAMTOR2*$_{del}$ mice were analyzed for the indicated cytokines by immune multiplexing. Two technical replications were performed. Mean±SD, *ns p>0.05, * p<0,05* as determined by unpaired Student's t-test. (**b**) Spleen cells of 3 control and CD11c *LAMTOR2*$_{del}$ mice were isolated. 5 x10^{6} cells were cultured overnight in R10 medium. Next day, supernatant was collected and cytokines were analyzed by multiplexing. Mean±SD, *ns p>0.05, * p<0,05, ** p<0,01* as determined by unpaired Student's t-test.

Fig. S4 **a**

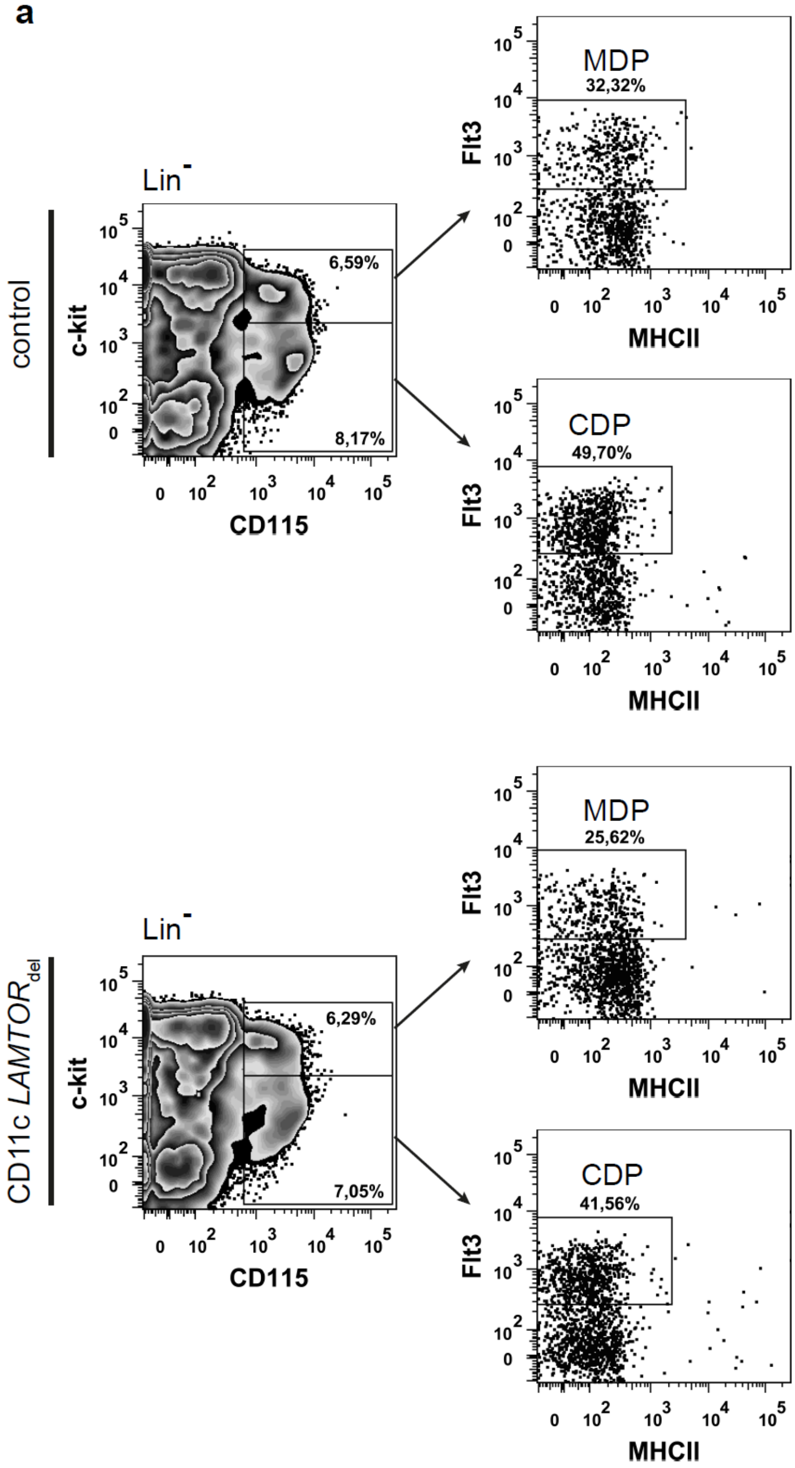

Supplementary Figure 4:

(**a**) Gating strategies for MDPs and CDPs in the BM by flow cytometry analyses. One representative example out of 4 individual measurements is shown.

Fig. S5

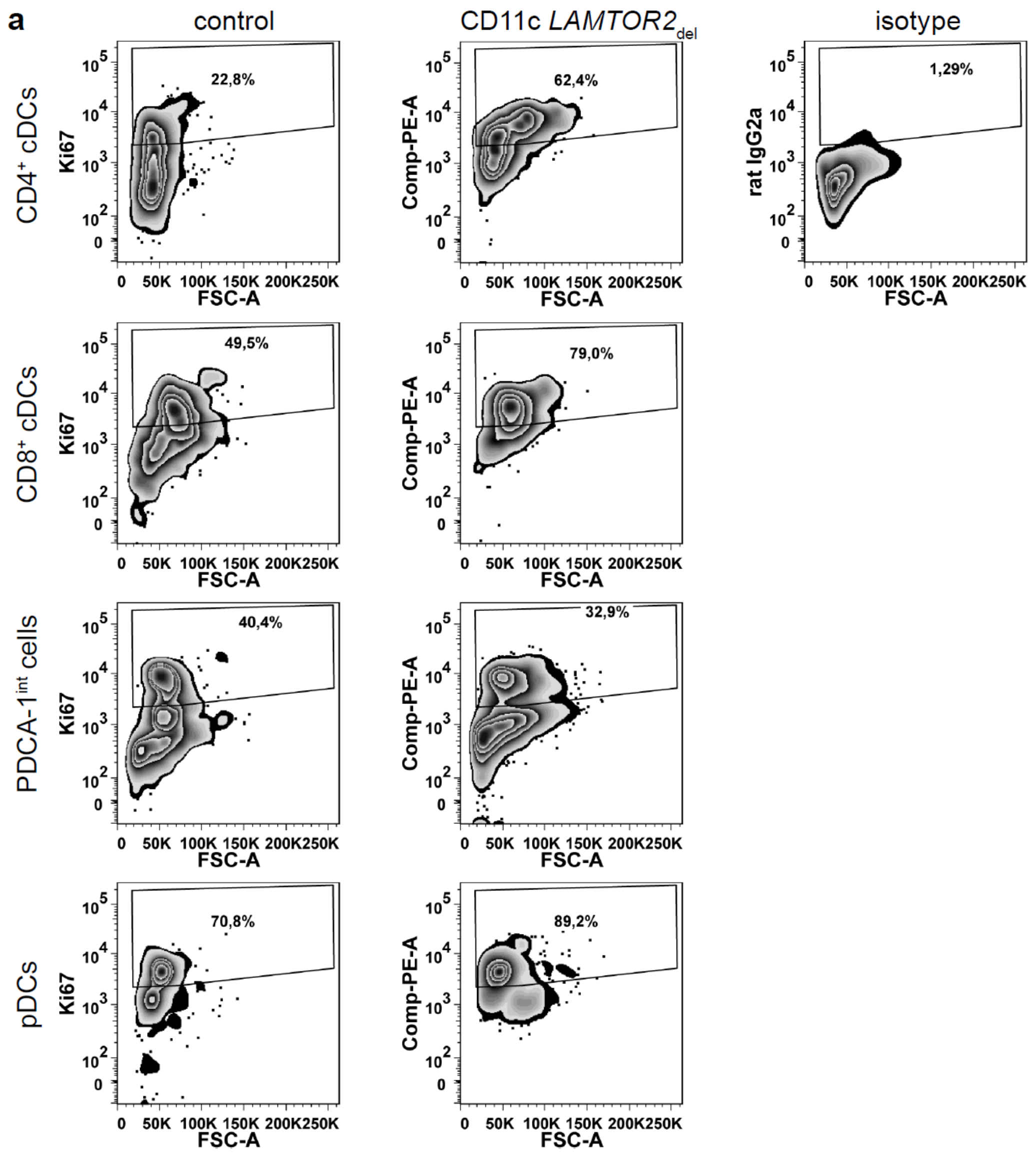

Supplementary Figure 5:

(**a**) Gating strategy for Ki67 positive cells. Cells were pre-gated for cDCs, pDCs and PDCA-1^{int} cells as shown in Fig. 1d and 2a and then gated for Ki67 according to the isotype control.

Fig. S6

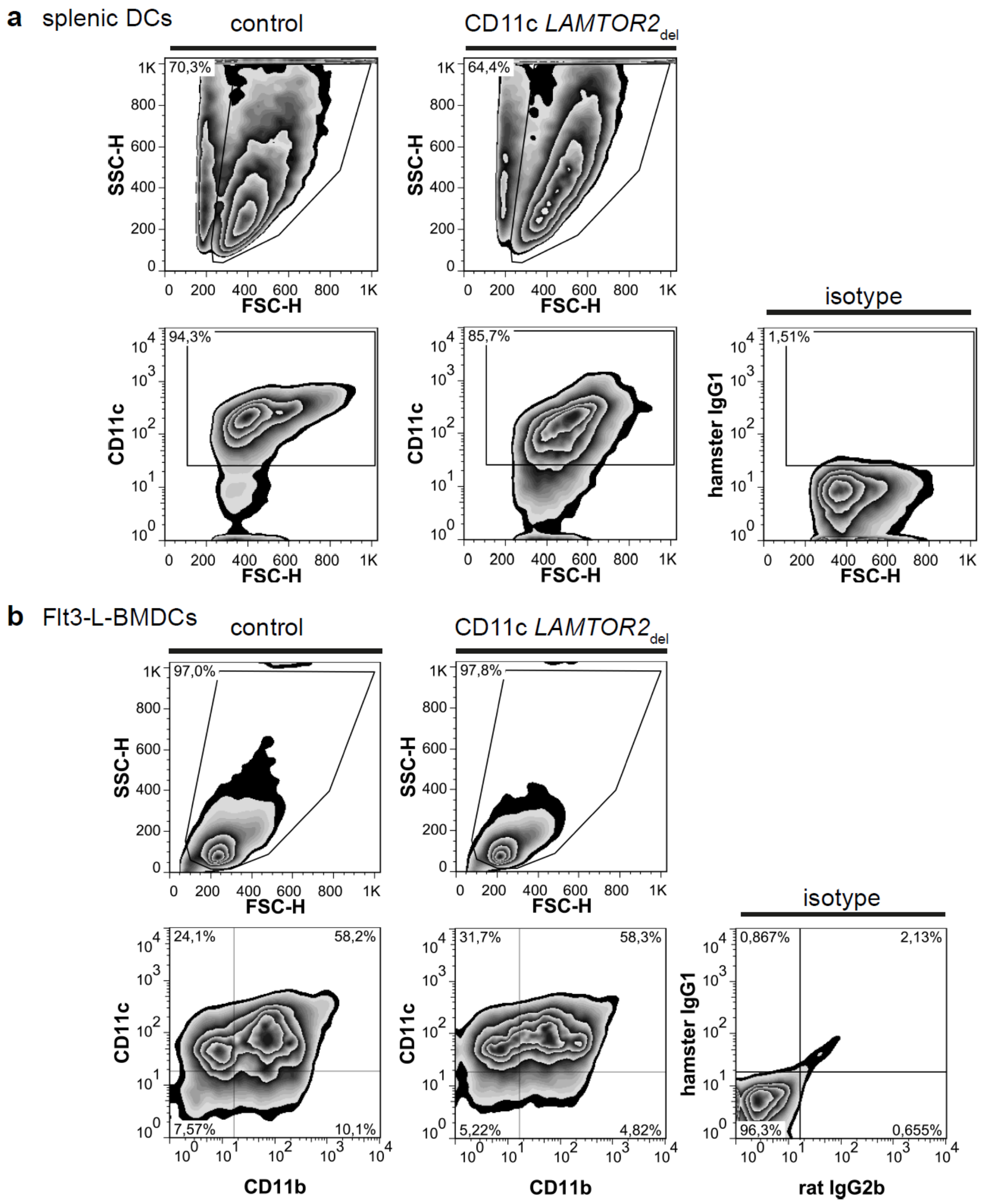

Supplementary Figure 6:

(**a**) Gating strategy for splenic DCs from control and CD11c *LAMTOR2*$_{del}$ mice after MACS preparation is shown. Cells were pre-gated by FSC and SSC and then gated for CD11c according to the isotype control. (**b**) BMDCs cultured in presence of FL were analyzed via flow cytometry for their purity. Cells were harvested on day 8 and viable cells were pre-gated by FSC and SSC. DCs are analyzed as CD11c^{+} CD11b^{+} and as CD11c^{+} CD11b^{-} cells according to the isotype controls.

Fig. S7

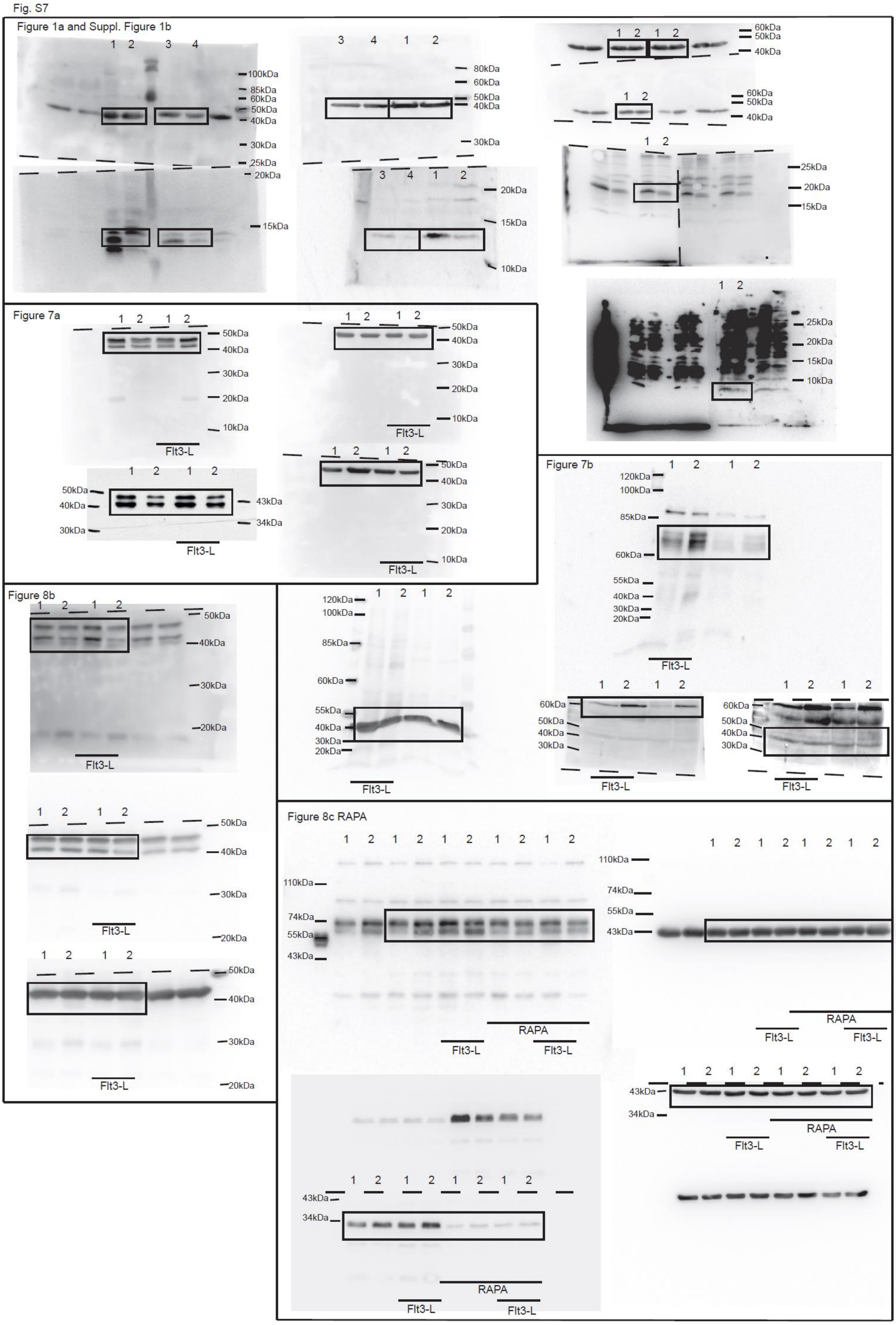

Supplementary Figure 7:

Shown are uncropped Western blots to the corresponding figures. Dotted lines indicate where the membranes were cut to allow incubation with different antibodies. M: marker, 1: control and 2: CD11c *LAMTOR2*$_{del}$ BMDC lysates, 3: control and 4: CD11c *LAMTOR2*$_{del}$ BMDC lysates. Treatments as indicated.

Fig. S8

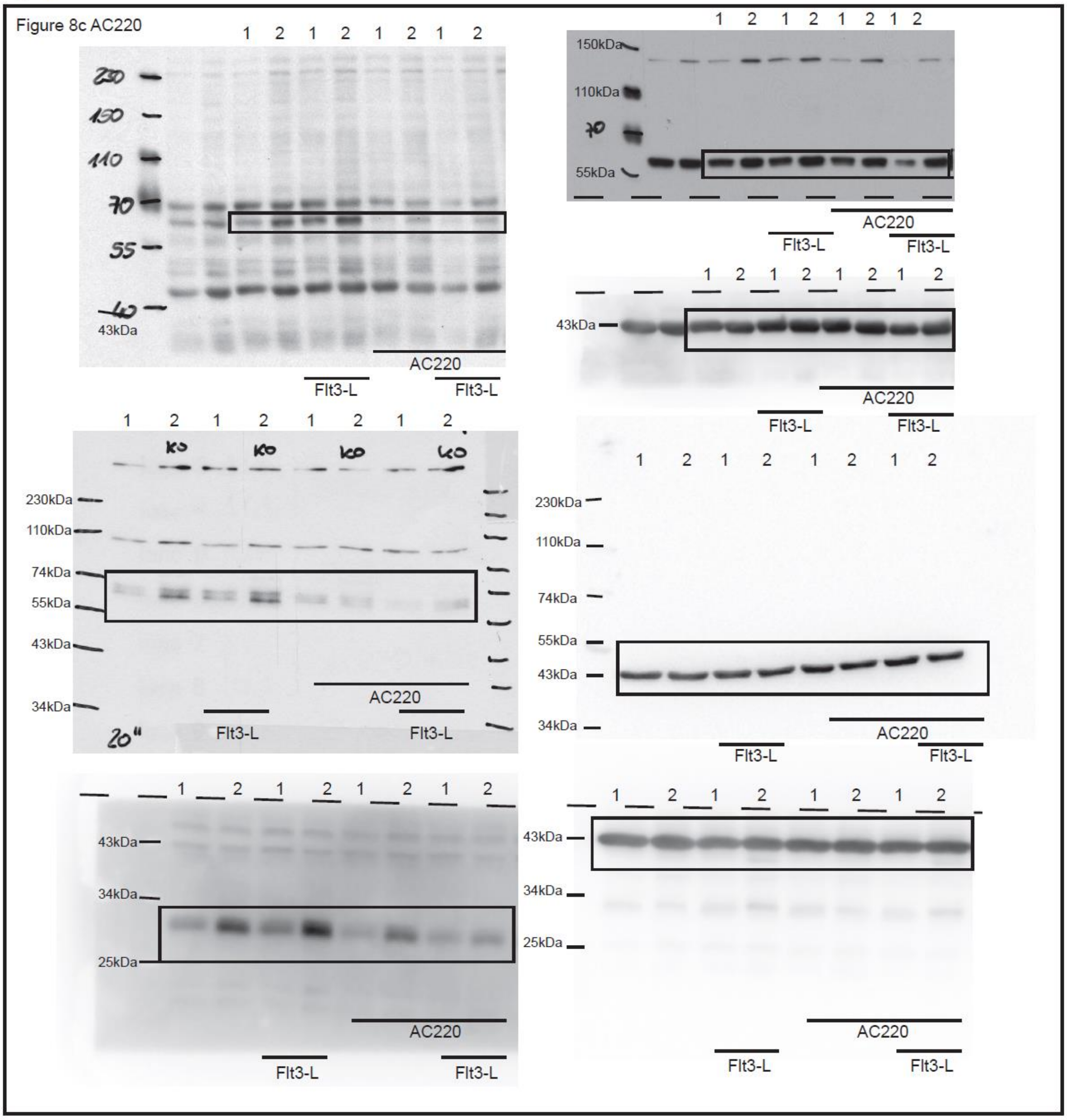

Supplementary Figure 8:
Shown are uncropped Western blots to the corresponding figures. Dotted lines indicate where the membranes were cut to allow incubation with different antibodies. M: marker, 1: control and 2: CD11c *LAMTOR2*$_{del}$ BMDC lysates, 3: control and 4: CD11c *LAMTOR2*$_{del}$ BMDC lysates. Treatments as indicated.
